# Interplay of lipid head group and packing defects in driving amyloid-beta–mediated myelin-like model membrane deformation

**DOI:** 10.1016/j.jbc.2023.104653

**Published:** 2023-03-27

**Authors:** Anuj Tiwari, Sweta Pradhan, Achinta Sannigrahi, Amaresh Kumar Mahakud, Suman Jha, Krishnananda Chattopadhyay, Mithun Biswas, Mohammed Saleem

**Affiliations:** 1 Department of Life Sciences, National Institute of Technology, Rourkela, India; 2 Department of Physics and Astronomy, National Institute of Technology, Rourkela, India; 3 CSIR - Indian Institute of Chemical Biology, Kolkata, India; 4 School of Biological Sciences, National Institute of Science Education and Research, Bhubaneswar, India; 5 Homi Bhabha National Institute, Mumbai, India; 6 Centre for Interdisciplinary Sciences, National Institute of Science Education and Research, Bhubaneswar, India

**Keywords:** amyloid beta, membrane, myelin-like model membrane, lipid-peptide interaction, neurodegeneration, Alzheimer’s disease

## Abstract

Accumulating evidence suggests that amyloid plaque-associated myelin lipid loss as a result of elevated amyloid burden might also contribute to Alzheimer’s disease. The amyloid fibrils are closely associated with lipids under physiological conditions; however, the progression of membrane remodeling events leading to lipid–fibril assembly remains unknown. Here we first reconstitute the interaction of amyloid Beta 40 (Aβ-40) with myelin-like model membrane and show that the binding of Aβ-40 induces extensive tubulation. To look into the mechanism of membrane tubulation, we chose a set of membrane conditions varying in lipid packing density and net charge that allows us to dissect the contribution of lipid specificity of Aβ-40 binding, aggregation kinetics, and subsequent changes in membrane parameters such as fluidity, diffusion, and compressibility modulus. We show that the binding of Aβ-40 depends predominantly on the lipid packing defect densities and electrostatic interactions and results in rigidification of the myelin-like model membrane during the early phase of amyloid aggregation. Furthermore, elongation of Aβ-40 into higher oligomeric and fibrillar species leads to eventual fluidization of the model membrane followed by extensive lipid membrane tubulation observed in the late phase. Taken together, our results capture mechanistic insights into snapshots of temporal dynamics of Aβ-40–myelin-like model membrane interaction and demonstrate how short timescale, local phenomena of binding, and fibril-mediated load generation results in the consequent association of lipids with growing amyloid fibrils.

Emerging evidence suggests that amyloid beta aggregates forming neuritic plaques may induce impairment of the myelin sheath as a result of myelin lipid loss (1). The presence of elevated levels of amyloid Beta 40 (Aβ-40) in cerebrospinal fluid as well as the white matter (2, 3, 4, 5) further supports the idea that myelin lipid loss might be one of the earliest pathological characteristics during Alzheimer’s disease (AD) progression (6). Despite the evidence that the soluble forms of Aβ-40 are elevated in the white matter, whether Aβ-40 aggregation can alter the myelin membrane remains elusive (3). Historically, although lipids have often been observed to associate with amyloid plaques, they were largely considered to be contaminants. A growing body of evidence suggests an active association of lipids during amyloid fibrillation (7). However, the mechanism that drives the progression of local lipid association during amyloid fibrillation remains unclear. The fibrillogenic properties of Aβ-40 and membrane damage have been observed to be significantly correlated (8, 9, 10, 11). Although both fibrils and oligomeric Aβ-40, unlike monomeric forms, were reported to induce a decrease in mitochondrial membrane potential in neurons, the presence of oligomeric forms in the mature fibril cannot be completely ruled out (12). Another recent report showed the binding of Aβ protofibrils to the liposome using Cryo-EM (13). A two-step mechanism has been proposed as a general mechanism behind membrane damage involving both the pore formation and fibril-mediated membrane deformation; however, the changes in membrane parameters and the transition from the early binding events to the late membrane deformation are missing (14, 15, 16). Thus, despite the body of reported work supporting that the amyloid cytotoxicity is majorly due to small oligomeric Aβ-40, the toxic peptide’s real nature is still a matter of debate (14). More recently, secondary nucleation mechanisms such as seeding that result in the acceleration of amyloid fibril formation by reducing or inhibiting the lag phase of fibril formation are now considered the major driving force in the progression of Aβ aggregation (17). The current understanding of amyloid-mediated membrane damage does not take into account the secondary nucleation aspect, thus making it important to investigate the same under secondary nucleation conditions as the presence and absence of seed significantly change the kinetics of aggregation.

Here we reconstitute *in vitro* the binding of seeded Aβ on the myelin-like model membrane which is made up of lipids that constitute a majority of the myelin membrane (18, 19) and visualize snapshots of the changes at the membrane interface over a period of early, mid, and late phases of Aβ aggregation spanning 24 h, using a combination of photonic and electron microscopy, fluorescence spectroscopy, and membrane monolayer experiments. Enhanced fluidization of the myelin membrane during early binding and elongation of Aβ was found to precede the extensive membrane tubulation (lipid association with fibril) observed in the late phase that also triggered fibril-mediated weak phase separation within the membrane. Dissection of the early binding of Aβ to lipid components of the myelin varying in their shape and charge revealed particularly high lipid specificity for 1,2-dioleoyl-sn-glycero-3-phospho-(1′-rac-glycerol) (DOPG), PI, sphingomyelin (Brain, Porcine) (BSM), and L-α-phosphatidylinositol-4,5-bisphosphate (PIP_2_) membranes. Upon observing the differential binding to different lipid constituents as well as the varying effect on the Aβ aggregation kinetics, we then set out with a hypothesis that given the complexity of the myelin membrane and the diverse shapes of its lipid components, can lipid defects play any role besides the electrostatic interactions? We then investigated the Aβ binding to membranes with an increasing level of lipid diversity in terms of shape, besides also quantifying the lipid defects densities through Coarse grain MD simulations. We show that the degree of binding of Aβ40 on membranes decreases in the following order: myelin-like model membrane (most defects and negative charge) > 1,2-dioleoyl-sn-glycero-3-phosphocholine/Brain Sphingomyelin/Cholesterol/L-α-phosphatidylinositol-4,5-bisphosphate (DOPC/BSM/Chol/PIP2) (fewer defects and negative charge) > DOPC/BSM/Chol (fewer defects without negative charge) > DOPC (more defects and zwitterionic) > DOPC/BSM/Chol/PG (negatively charged but inverted conical PIP2 replaced by a cylindrical PG). The binding of Aβ-40 depends predominantly on the lipid packing defect densities and electrostatic interactions and results in rigidification of the myelin-like model membrane during the early phase of amyloid aggregation evident from the reduction in lipid diffusion. Furthermore, elongation of Aβ-40 into higher oligomeric and fibrillar species leads to eventual fluidization of the model membrane followed by extensive lipid membrane tubulation observed in the late phase suggesting lipid association with the growing fibril. Together, our results capture mechanistic insights into snapshots of temporal dynamics of Aβ-40–myelin membrane interaction and demonstrate how lipid packing densities and electrostatics of the membrane interfaces drive binding. Further, our work also provides insights into the modulation of membrane parameters by the fibril-mediated load generation that results in the lipid association with growing fibril.

## Results

### Binding and deformation of myelin membrane by Aβ-40

**Figure 1:**
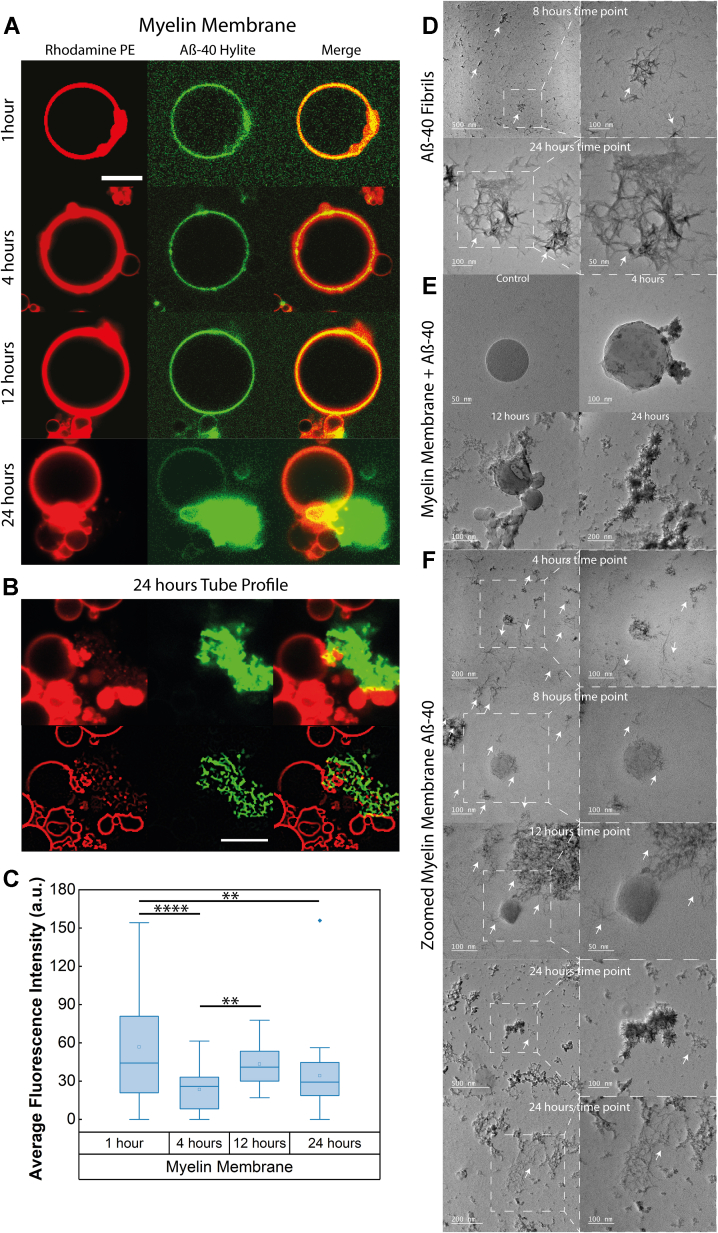
**Binding and deformation of myelin-like model membrane by Aβ-40.***A*, temporal changes in the morphology of the GUV equatorial contour observed in confocal imaging observed at 1, 4, 12, and 24 h with representative images shown from a pool of GUVs at each time point. GUVs are labeled with Rhodamine PE (*red channel*) and incubated with Aβ-40 doped with Hylite-488 Aβ-40 (*green channel*) under evaporation-free conditions to monitor the changes in the membrane during the Aβ-40 aggregation phase. *B*, tubeness profile of the membrane morphology induced by Aβ-40 at late aggregation phase. *C*, average fluorescence intensity of the Aβ-40 binding at the equatorial plane of GUV observed at 1, 4, 12, and 24-h time points. The number of GUVs screened in the box plot is n = 35 from three independent experiments. The symbols ∗∗ and ∗∗∗∗ indicate *p* values of ≤0.01 and 0.0001, respectively, calculated by one-way ANOVA followed by Bonferroni's multiple comparison test. *D*, TEM micrographs of Aβ-40 aggregation at 8 and 24 h with a marked inset for the zoomed images on the right. *E*, TEM micrographs of temporal changes in the membrane morphology induced by Aβ-40 aggregation imaged over 24 h. *F*, TEM micrographs of temporal changes in the membrane morphology induced by Aβ-40 aggregation imaged over 24 h with a marked inset for the zoomed images on the *right*. *White arrows* in the TEM micrographs mark the fibrils. The scale bar for confocal microscopy images is 10 μm. Aβ 40, amyloid Beta 40; GUV, giant unilamellar vesicles.

The inclusion of lipids with Aβ fibrils and plaques resulting in neuronal membrane lipid loss is often observed *in vivo*. Similarly, recent *in vitro* studies demonstrated that Aβ oligomer triggered substantial lipid loss in membranes mimicking the extracellular face of the cell membrane (20). However, the quantitative aspects of membrane modulation by Aβ leading to lipid extraction are lacking as highlighted by a very recent review (7). To examine whether the Aβ-40 interaction could trigger the deformation of the myelin membrane, we sought to use the reconstitution methodology to allow us to mimic the myelin membrane exposed to a bulk concentration of soluble Aβ-40 and map any morphological changes in the membrane over different phases of Aβ-40 aggregation. To this end, giant unilamellar vesicles (GUVs) were reconstituted using the majority lipid components of the myelin membrane to mimic the outermost bilayer of the myelin sheath membrane composed of DOPC/BSM/DOPE/PI/DOPS supplemented with cholesterol (18). A limitation of the chosen composition is that cerebrosides and sulfatides could not be incorporated as they are known to destabilize the bilayer, particularly when present in complex mixtures, and induce the formation of highly curved non-lamellar structures or nanoscopic flat stacked disk-like structures (21). The chosen composition reasonably mimics the complexity of lipid components of the myelin membrane, in terms of both the topological aspects of the lipids (*i.e.*, a mixture of cylindrical, conical, and inverted conical lipids with varying hydrophobic volumes) and surface charge (Figs. 1 and S2). We pre-seeded Aβ-40 with oligomeric-Aβ-40 before adding it to the membrane and monitored over 1, 4, 12, and 24 h to mimic the physiologically relevant secondary nucleation mechanisms (22). To quantify different populations of soluble forms of Aβ-40, we measured the hydrodynamic radii extracted through the diffusion coefficients determined by fluorescence correlation spectroscopy, wherein the monomer-to-oligomer ratio of 9:1 was observed (Fig. S1 and Table S1). From here on, throughout the article, the early phase refers to the 0 to 4 h time point, the mid-phase refers to the 4 to 12-h time point, and the late phase refers to the 12 to 24-h time point for ease of understanding.

Aβ-40 showed significant binding to the myelin-like model membrane starting early phase (*i.e.*, visualized at 1 h) as evident from the binding intensity, followed by a decrease in the binding at 4-h time point. The early phase likely involves the binding of the population of Aβ-40 that is predominantly monomeric to lower oligomeric. However, the binding of protofibrils cannot be ruled out as it is technically challenging to quantify the same in the bound state, given the highly dynamic state of aggregation. At the 12-h time point again, an increase in the binding intensity was observed (Fig. 1*C*). Interestingly, at 24 h, Aβ-40 induced striking membrane deformation at the interface, as evident from the tubulation profile of the distorted membrane regions (Fig. 1*B*), also accompanied by clustering of vesicles (Fig. S12). The same was observed on the supported lipid bilayer starting 8 to 12 h (Fig. S2*B*). From the observed overlay of lipid and Aβ channel, it is likely elongating Aβ-40 fibril extracts lipids into tubular structures. Further, negative staining electron microscopy of the myelin-like model membrane incubated with Aβ-40 over a time period of 4, 12, and 24 h confirmed the fibrillar network as well as the progression of Aβ-40–induced disruption of the model membrane (Fig. 1, *A*, *B*, *E*, and *F*). Moreover, Aβ-40 aggregation is known to be enhanced by curvature, and therefore, it might be reasonable to believe that these are indeed lipid membrane tubules, possibly enriched in DOPE, extracted by the growing Aβ-40 fibrils (23).

### The interplay of lipid specificity and fibrillation of Aβ-40

**Figure 2:**
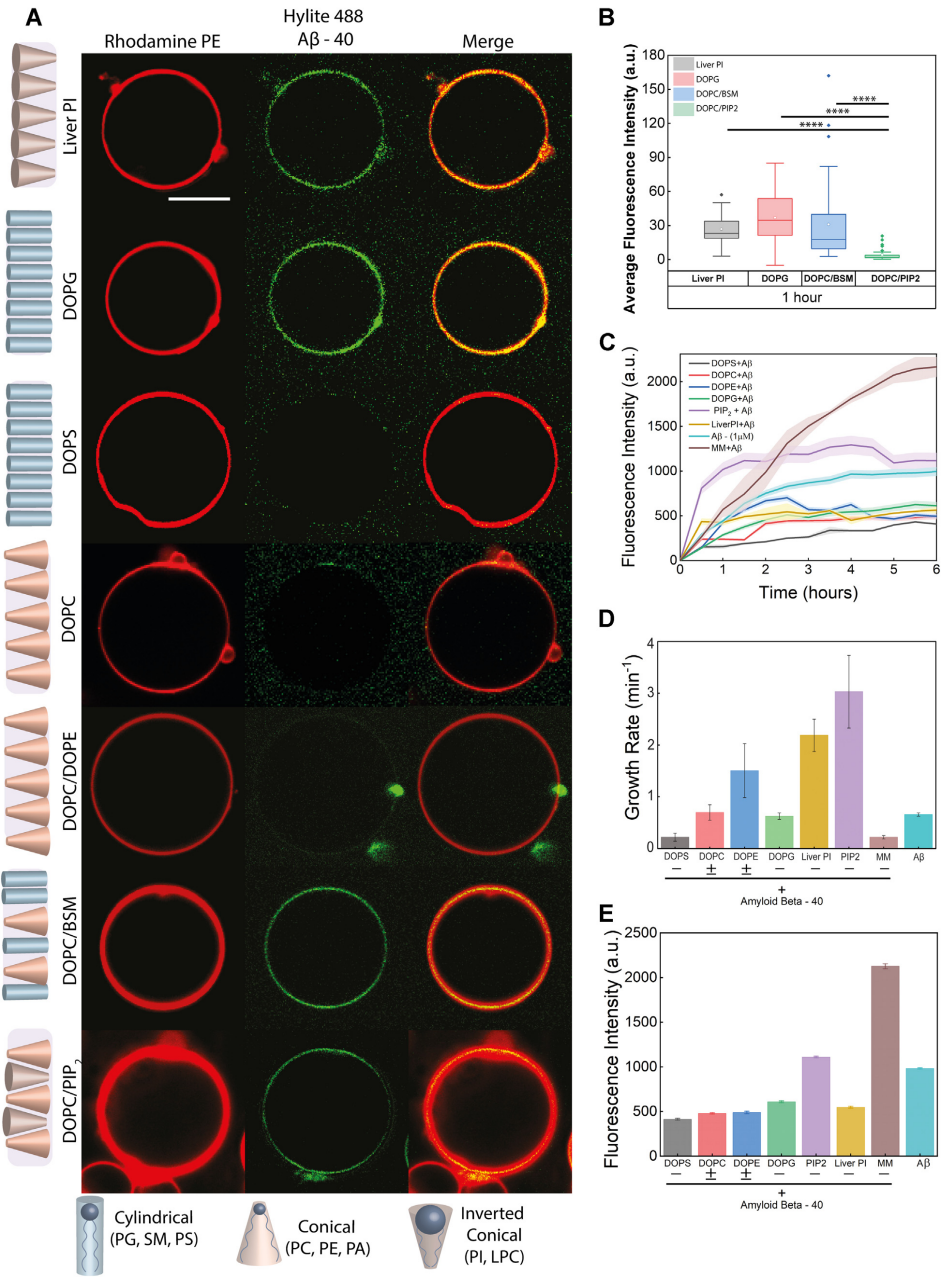
**Lipid specificity of the Aβ-40 early binding and fibrillation.***A*, GUVs labeled with Rhodamine PE (*red channel*) were incubated with Aβ-40 doped with Hylite-488 Aβ-40 (*green channel*) to monitor the binding of the peptide within 1 h (early phase). Early binding of Aβ-40 was observed in the case of PI, DOPG, DOPC/BSM, and DOPC/PIP2 membranes. No binding of Aβ-40 is seen on DOPS and DOPC/DOPE membranes. *B*, average fluorescence binding intensity of Aβ-40 on membranes with different head groups. The number of GUVs screened for each condition in the box plots is n = 35 from three independent experiments. The symbol ∗∗∗∗ indicates *p* values of ≤0.0001, calculated by one-way ANOVA followed by Bonferroni's multiple comparison test. *C*, ThT fluorescence assay to monitor the effect of lipid head groups on the aggregation kinetics of Aβ-40. *D*, effect of lipid head groups on the growth rate (change in fluorescence intensity units per minute) of Aβ-40 aggregation quantified within the early phase of aggregation time scales *i.e.*, 30 min). *E*, effect of lipid head groups on the sustained aggregation of Aβ-40 during the late phase (saturation phase) of aggregation kinetics, *i.e.*, 6 h). ± refers to the net charge on the membrane (*i.e.*, negative or zwitterionic). The scale bar for confocal microscopy images is 10 μm. Aβ 40, amyloid Beta 40; BSM, sphingomyelin (Brain, Porcine); DOPC, 1,2-Dioleoyl-sn-glycero-3-phosphocholine; DOPE, 1,2-dioleoyl- sn-glycero-3-phosphoethanolamine; DOPG, 1,2-dioleoyl-sn-glycero-3-phospho-(1′-rac-glycerol); DOPS, 1,2-dioleoyl-sn-glycero-3-phospho-L-serine; DPPC, 1,2-dipalmitoyl-sn-glycero-3-phosphocholine; liver PI, L-α-phosphatidylinositol; PIP2, L-α-phosphatidylinositol-4,5-bisphosphate.

Considering that several lipid components constitute the myelin membrane, we next asked whether there is specificity for diverse lipid geometry and the head group charge that could play an important role in dictating Aβ-40 binding and aggregation. The shape of lipids depends on the aspect ratio of their headgroups and acyl chains that determine their packing, the degree of local defects, and spontaneous curvature within the membrane (24). To evaluate this, we first focused on the early phase of its interaction and aggregation corresponding to a time scale of the first hour. Interestingly, while no significantly visible early binding of Aβ-40 was observed in the DOPC membrane (conical/zwitterionic lipid) (25, 26)) in the first hour (Fig 2*A*), homogenous binding was observed in the case of DOPG (cylindrical/negatively charged lipid) and PI membrane (inverted conical/negatively charged lipids) (Fig. 2*A* and S3).

Further, no binding was observed in the DOPC/DOPE (3:2) membrane composed of both cylindrical and conical lipids with an overall neutral charge (27) (Fig. 2*A*). Surprisingly, Aβ-40 was found to bind the DOPC/BSM (3:2) membrane composed of conical and cylindrical lipids but with different acyl chain length (hydrophobic mismatch) bearing a neutral charged more intensely, compared to the DOPC/PIP2 (3:2) membrane composed of both conical and inverted conical lipids with a net negative charge (Fig. 2*A*). We further examined the influence of the constituent lipid components (DOPC, DOPE, DOPS, PIP2, PI, DOPG, and myelin-like model membrane) on aggregation kinetics of Aβ-40 by incubating 1 μM Aβ-40 with membranes composed of 200 μM of different lipid membranes. Examining the growth rate measured for the initial surge in the fluorescence intensity observed within a time window of 30 min, among all the lipid membranes, PIP2 triggered the fastest fibril formation followed by PI, both of which are inverted conical in shape and negatively charged (Fig. 2, *C* and *D*). The zwitterionic conical DOPC and negatively charged cylindrical DOPG were found to have no significant effect on fibrillation in the early phase kinetics as evident from extracted growth rates (Fig. 2*D*). However, looking at the overall aggregation kinetics, at late or saturated phase, DOPC tends to slow down the Aβ-40 aggregation (Fig. 2, *C* and *E*).

Interestingly, despite the early binding of Aβ-40 on the reconstituted myelin-like model membrane, the observed growth rate was the lowest among all, hinting at slow fibrillation (Fig. 2*D*). The model membrane was also found to facilitate Aβ-40 fibrillation most strongly and sustain it over a longer duration, as evident from the ThT fluorescence intensity observed at the plateau (Fig. 2*E*). Among all the membranes, only PIP2 was found to facilitate fibrillation as evident from higher plateau (Fig. 2*E*). The differential binding of Aβ-40 to single and binary lipid compositions such as between DOPC & DOPS (No early binding of Aβ) and DOPC/BSM, DOPC/PIP2 (early binding of Aβ was observed) were intriguing and led us to conclude there are more parameters involved besides the electrostatic interactions that might be important for the binding. And one such parameter that was strikingly different for different components of the myelin mimic was the lipid shape. Thus, we set out with a hypothesis that given the complexity of the myelin membrane and the diverse shapes of its lipid components, can lipid defects play any role besides the electrostatic interactions in myelin membrane deformation?

### Myelin membrane contains higher lipid packing density defects

**Figure 3:**
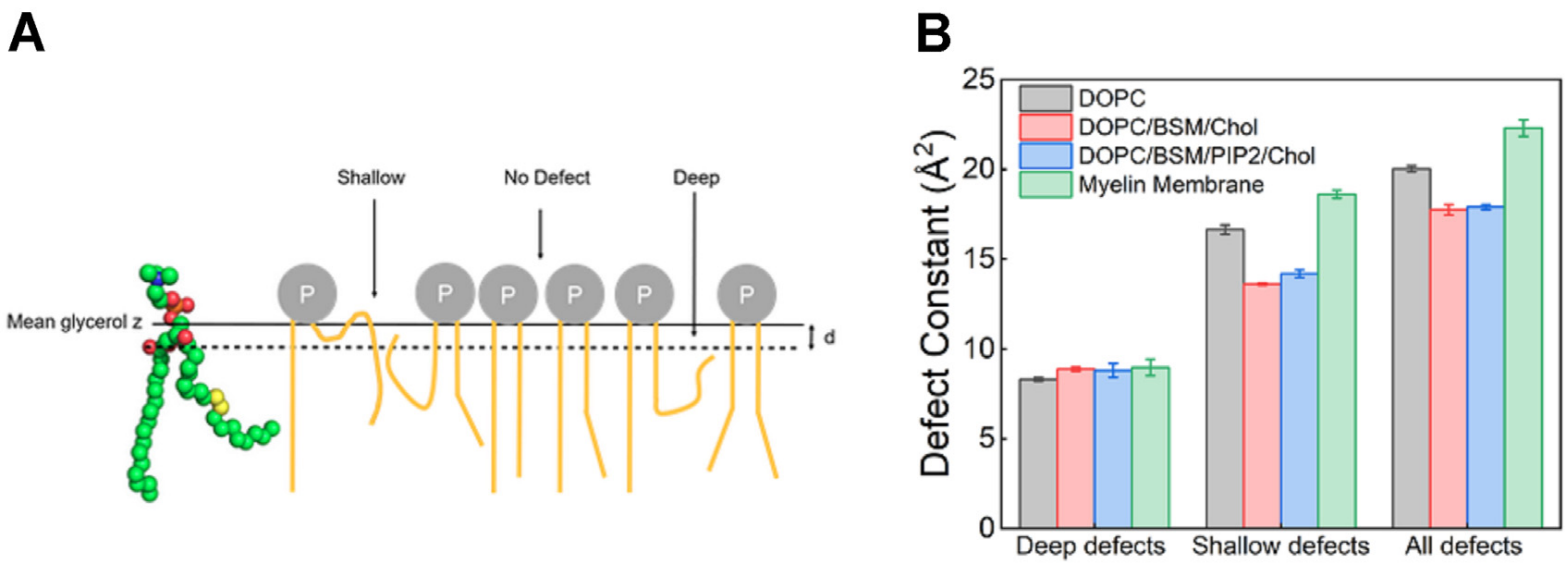
**Simulation-based quantification of the lipid packing defect constants.***A*, an illustration to provide a lateral view of shallow, deep, and no defects at the membrane surface. *B*, histogram of the defect constant for deep, shallow, and all (both deep and shallow defects) defects for each of the four conditions, namely, DOPC (*light grey*), DOPC/BSM/Chol (*light red*), DOPC/BSM/PIP_2_/Chol (*light blue*), myelin-like model membrane (*light green*). BSM, sphingomyelin (Brain, Porcine); DOPC, 1,2-Dioleoyl-sn-glycero-3-phosphocholine; GUV, giant unilamellar vesicles; PIP2, L-α-phosphatidylinositol-4,5-bisphosphate.

To test our hypothesis, we designed four different membrane conditions varying in lipid geometry, hydrophobic volumes, and net charge and quantified the lipid packing defect density using used coarse-grained molecular dynamics simulation. The following four membranes were adopted to mimic different degrees of lipid packing defects: (i) zwitterionic conical lipids (DOPC), (ii) zwitterionic conical and cylindrical lipids (DOPC/BSM/Chol), (iii) negatively charged inverted conical, zwitterionic conical, and cylindrical lipids (PIP2/DOPC/BSM/Chol), and (iv) a more complex surface containing negatively charged inverted conical, zwitterionic conical, and cylindrical lipids (myelin-like model). We used PackMem (28) to quantify the lipid packing defects that follow the Cartesian grid system for mapping the membrane surface where the grid dimension is set to 1 Å × 1 Å. Using PackMem execution upon each of the membrane systems, we produced a plot comparing the value of defect constant (π) for the three types of defects (deep, shallow, and all) shown in Figure 3 (please see Experimental procedures for details of CGMDS). The higher the π constant, the more abundant and larger the packing defects (28). Indeed, of the four bilayer systems studied, the reconstituted myelin-like model membrane has more numbers of packing defects, and out of deep and shallow defects, shallow defects are more abundant. The scale of the observed variation in the lipid packing defect densities of chosen membrane surfaces is in the range of 3 to 5 Å and holds significant relevance in the biological regime.

### The interplay of lipid packing defects and electrostatics drives Aβ-40 fibrillation and membrane deformation

**Figure 4:**
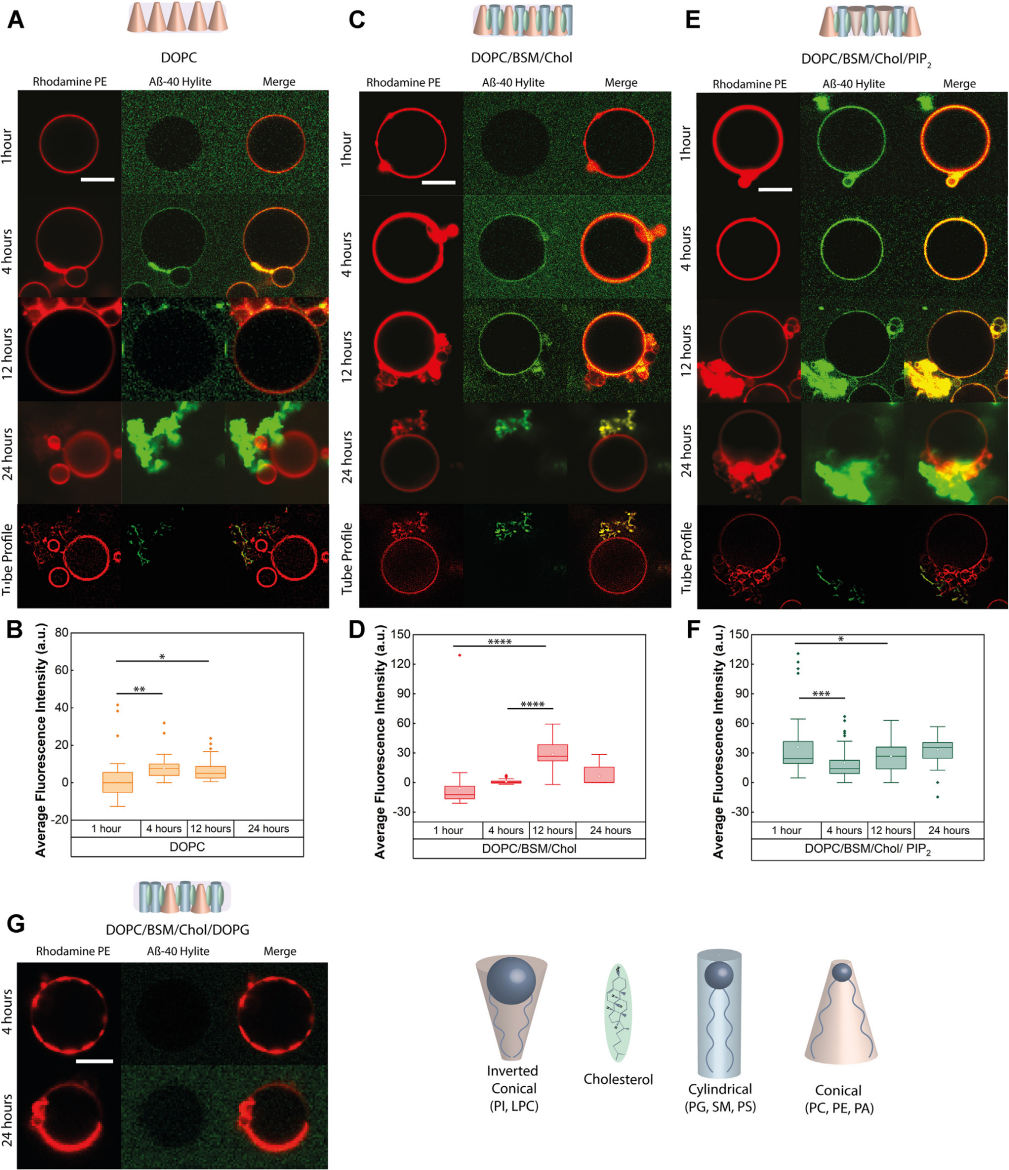
**Effect of lipid packing defects and electrostatics on Aβ-40 binding and membrane deformation.***A*, GUVs of DOPC lipid doped with 1% Rhodamine PE (*Red channel*) incubated with Aβ-40 doped with Hylite-488 Aβ-40 (*Green channel*) and monitored temporally at 1, 4, 12, and 24 h for changes in binding intensity. Tube profile was extracted at the 24-h time point to visualize the deformations induced by Aβ-40 over time. *B*, *box plot* showing weak to no binding of Aβ-40 to the DOPC GUV population observed at an early time point (1 h), whereas, at later time points the binding increased and reached a plateau. *C*, temporal monitoring of GUVs of DOPC/BSM/Chol lipid in the ratio 4:4:2 doped with 1% Rhodamine PE (*Red channel*) and incubated with Aβ-40 doped with Hylite-488 Aβ-40 (*Green channel*) at 1, 4, 12, and 24 h for changes in binding intensity. The tube profile was extracted at the 24 h time point to visualize the deformations induced by Aβ-40 over time. *D*, no binding was seen at the early time point (1 h) for the DOPC/BSM/Chol (4:4:2) GUV population, with a steady rise in intensity at later time points. *E*, GUVs of DOPC/BSM/Chol/PIP_2_ lipids in the ratio 2:4:3:1 doped with 1% Rhodamine PE (*Red channel*) incubated with Aβ-40 doped with Hylite-488 Aβ-40 (*Green channel*) and monitored temporally at 1, 4, 12, and 24 h for changes in binding intensity. The tube profile was extracted at the 24-h time point to visualize the deformations induced by Aβ-40 over time. *F*, box plot of the binding intensity of Aβ-40 to the GUV population of DOPC/BSM/Chol/PIP_2_ (2:4:3:1) showing a strong early binding sustained till longer time points. *G*, temporal monitoring of DOPC/BSM/Chol/DOPG (2:4:3:1) GUVs doped with 1% Rhodamine PE (*Red channel*) incubated with Aβ-40 doped with Hylite-488 Aβ-40 (*Green channel*) at 4 and 24 h showed no binding at early and late time points. The number of GUVs screened at each time point for each condition in the *box plots* is n = 35 from three independent experiments. The symbols ∗, ∗∗, ∗∗∗, and ∗∗∗∗ indicate *p* values of ≤0.05, 0.01, 0.001, and 0.0001, respectively, calculated by one-way ANOVA followed by Bonferroni’s multiple comparison test. The scale bar for confocal microscopy images is 10 μm. BSM, sphingomyelin (Brain, Porcine); DOPC, 1,2-Dioleoyl-sn-glycero-3-phosphocholine; GUV, giant unilamellar vesicles; PIP2, L-α-phosphatidylinositol-4,5-bisphosphate.

To validate our hypothesis experimentally, we next questioned if the observed differences in the lipid packing defect densities might be the driving forces for the degree of early binding and subsequent deformation by Aβ-40. Since Aβ-40 aggregation is a slow process, we monitored the fate of Aβ-40 binding and changes induced in membrane morphology on a time scale of 24 h and captured the snapshots at early (1–4 h), mid (4–12 h), and late aggregation phases (12–24 h) (*i.e.*, at 1 h, 4 h, 12 h, and 24 h respectively) (Figs. 4 and S3–S5). We observed that in the case of DOPC, Aβ-40 was weakly membrane-bound at 4 h with no microscopically visible deformation of the membrane, as evident from the contour on the equatorial plane of the GUV (Fig. 4). Striking deformation of the membrane into large tubular structures was observed at 24 h, followed by visualization of a pool of GUVs that either have intense membrane tubulation or completely ruptured membrane in the late phase (around 24 h) (Fig. 4, *A* and *B*).

We next investigated the binding of Aβ-40 to the membrane composed of DOPC, BSM, and Cholesterol (4:4:2), accommodating a relatively lesser amount of lipid packing defects compared to the DOPC membrane. Interestingly, negligible to weak binding of Aβ-40 to DOPC/BSM/Chol membrane was observed in the early phase followed by increased binding and mild tubulation during the mid-phase (Fig. 4, *C* and *D*). This is unlike the DOPC/BSM membrane, which showed early binding of Aβ-40 (Fig. 1*A*). Similar to the DOPC membrane, a pool of tubulating and collapsed membrane was observed at late phase (24 h) (Fig. 4, *C* and *D*), although the tubulation was not as strong as in the case of the DOPC membrane (Fig. 4*A*). We reasoned that the presence of cholesterol is expected to increase the lipid packing density and stiffen the membrane containing unsaturated lipids, thereby reducing the membrane tubulation (29). To understand the coupling between lipid packing defects and electrostatic forces, we added a negatively charged inverted conical lipid PIP2 component to the DOPC/BSM/Chol membrane. Strong binding and tubulation mediated by Aβ-40 were observed in the presence of PIP2 during the early to mid-phase, although relatively excess cholesterol was present in the membrane (Fig. 4, *E* and *F*). Interestingly, no binding of Aβ-40 was observed from the early to the late phase when PIP2 in the DOPC/BSM/Chol/PIP2 membrane was replaced with DOPG (a negatively charged cylindrical lipid) that results in a reduction in lipid packing defects (Fig. 4*G*). The hydrogen bonding between glycerol moiety of DOPG and the phosphate oxygen of the neighboring phospholipid which leads to the ordering of the membrane might explain the reduction in the defect densities (30). We think that though early membrane binding of Aβ-40 could be predominantly driven by the lipid packing defects and the limiting bulk peptide concentration, however, the polar interactions are essential for the stabilization of the interactions. This is evident from the lack of early binding of Aβ-40 to DOPC which has significant lipid packing defects but not sufficiently strong electrostatic forces (Figs. 3 and 4*A*). In summary, comparing the membrane binding induced by Aβ-40 over time, the degree of binding and deformation on membranes by Aβ-40 decreases in the following order: myelin-like model membrane (cylindrical lipids with height mismatch, inverted conical and conical lipids) > DOPC/BSM/Chol/PIP2 (conical, cylindrical, inverted conical lipids) > DOPC/BSM/Chol (conical and cylindrical lipids) > DOPC (conical lipids) (Box plot, Figs. S6–S9). Together, the abovementioned observations suggest that the early binding and fibrillation of Aβ-40 depends on the interplay of both lipid geometry (that defines local lipid packing defects) and electrostatics. However, lipid packing defects could be the predominant factor among the two that dictates both the kinetics of Aβ-40 binding and membrane deformation.

### Contribution of lipid shape in the absence of charge for Aβ-40 binding

We then looked into the role of different lipid shapes (*i.e.*, a ratio of cones to cylinder) and cholesterol, in the absence of negative charge, that might affect the binding of Aβ-40. To test this, we modulated the ratios of DOPC (conical lipid), BSM (cylindrical lipid), and cholesterol (Fig. S10). We observed significant early binding of Aβ-40 on DOPC/BSM membranes in the absence of cholesterol both at a ratio of 5:5 as well as 3:2 (Figs. S10 and 2). Interestingly the binding efficiency of Aβ-40 was found to vary as the proportions of conical/cylindrical lipid changed, suggesting that not only the lipid shape is important but also the proportions of different lipid shapes might influence the lipid packing defects and subsequent binding of Aβ-40. Evidently, in the presence of an equal proportion of cholesterol, the reversal of the ratios of DOPC: BSM from 2:6 to 6:2 results in a significant weakening of the Aβ-40 early binding on the membrane (Fig. S10). Interestingly, even for the DOPC/BSM/Cholesterol ratio of 3:3:4 with double the proportion of cholesterol, early binding of Aβ-40 is observed to the liquid-disordered regions of the phase-separated membrane (Fig. S10). This further reinforces our hypothesis that lipid geometry that dictates packing defects contributes predominantly to the initial binding of Aβ-40.

### Aβ-40 drives myelin deformation through its fluidization

We reasoned that the observed differences in early and late binding of Aβ-40 should also reflect in the fluidity changes in the membrane. Irrespective of the density of lipid packing defects, Aβ-40 seems to bind to most membrane conditions by the 4-h time points (early to mid-phase) (Fig. 4). Thus, we next probed the fluidity changes in the membrane around 4 h, owing to the aggregation of Aβ-40. Steady-state fluorescence anisotropy measurements report the global changes in the fluidity of the lipid membrane. The changes in membrane fluidity were quantified by using a fluorescent probe TMA-DPH which generally incorporates into the polar region of the membrane (31). We also found that the overall trend remained largely unchanged till 8 h (Fig. S11).

**Figure 5:**
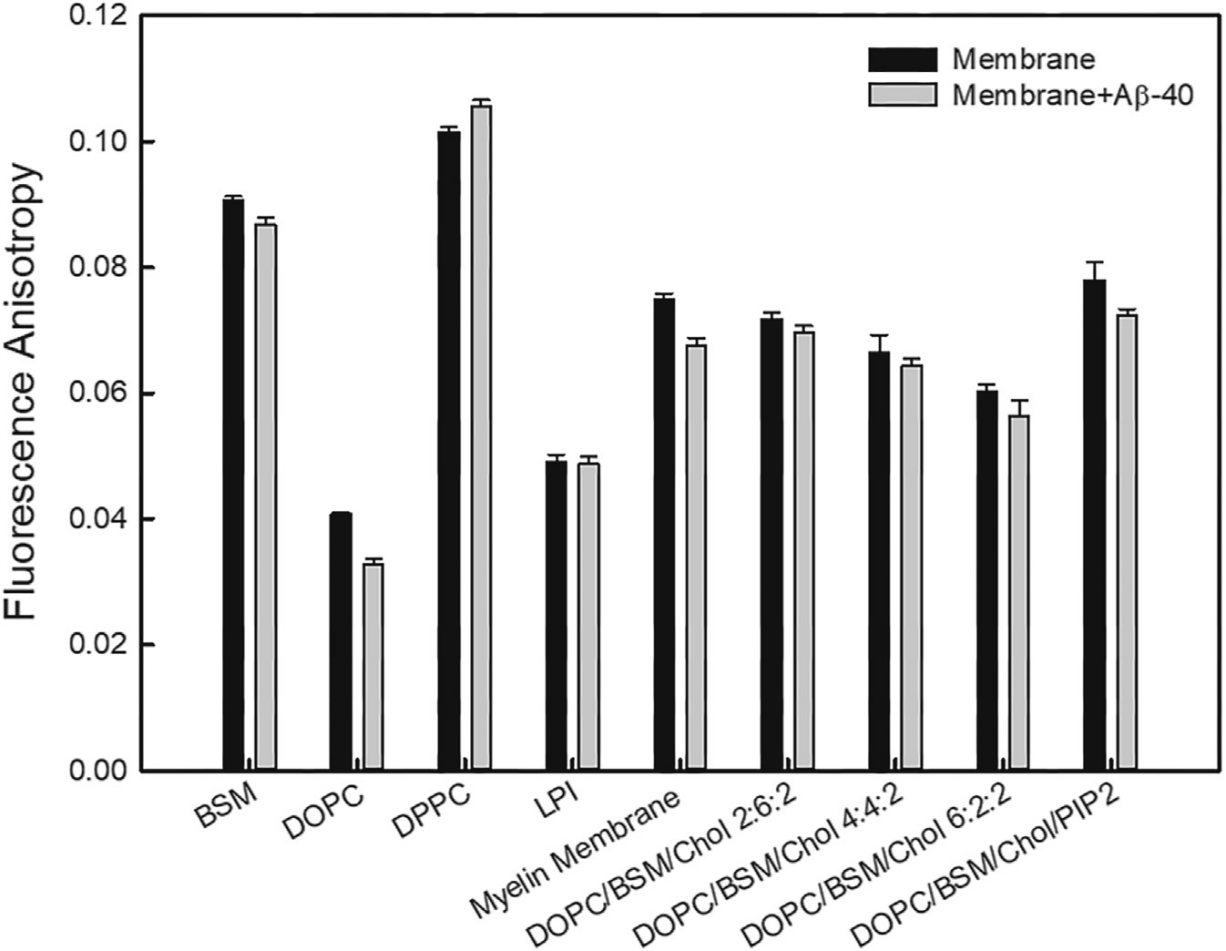
**Changes in membrane fluidity by Aβ-40.** Steady state anisotropy measurements were performed on different membranes probed with TMA-DPH. The following membrane conditions were investigated: single lipid membrane compositions like DOPC, BSM, LPI, DPPC, DOPC/BSM content keeping cholesterol constant (2:6:2, 4:4:2, 6:2:2), and multicomponent membranes of the myelin-like model membrane and DOPC/BSM/Chol/PIP_2_ in the presence of Aβ-40. The bar graphs represent the mean and standard deviation extracted from three independent experiments. BSM, sphingomyelin (Brain, Porcine); DOPC, 1,2-Dioleoyl-sn-glycero-3-phosphocholine; DOPS, 1,2-dioleoyl-sn-glycero-3-phospho-L-serine; DPPC, 1,2-dipalmitoyl-sn-glycero-3-phosphocholine; GUV, giant unilamellar vesicles; PIP2, L-α-phosphatidylinositol-4,5-bisphosphate.

We observed that the binding of Aβ-40 to the reconstituted myelin-like model membrane as well as all other membrane conditions, except DPPC and PI, results in a reduction in the fluorescence anisotropy of the probe, indicating an increase in fluidization (Fig. 5). The observed differences in the fluidity in the case of ternary conditions of DOPC/BSM/Chol could be attributed to both phase redistribution as well as protein-mediated changes in the membrane. We observed that the fluidity of PI membrane remains unchanged. To further confirm the correlation between lipid packing density, Aβ-40 binding, and subsequent increase or decrease in fluidization, we checked the effect of Aβ-40 binding on the fluidity of DPPC (saturated membrane). We observed a significant reduction in the fluidity of the DPPC membrane was observed. This is in support of a previous study wherein it was shown that the gel phase rigid domains of DPPC may act as a platform for Aβ enrichment that might further decrease the fluidity of the membrane (32). Taken together, sustained binding and fibrillation of Aβ-40 during the mid-phase results in fluidization of the membrane as evident from fluorescence spectroscopy shown in Figure 5. This leads us to conclude that albeit the shape and charge of the lipids in the membrane play an important role in the early binding of the peptide, the process progresses to subsequent disruption through fluidization of the membrane.

### Dynamics of lipid and Aβ-40 diffusion during Aβ-40 growth

**Figure 6:**
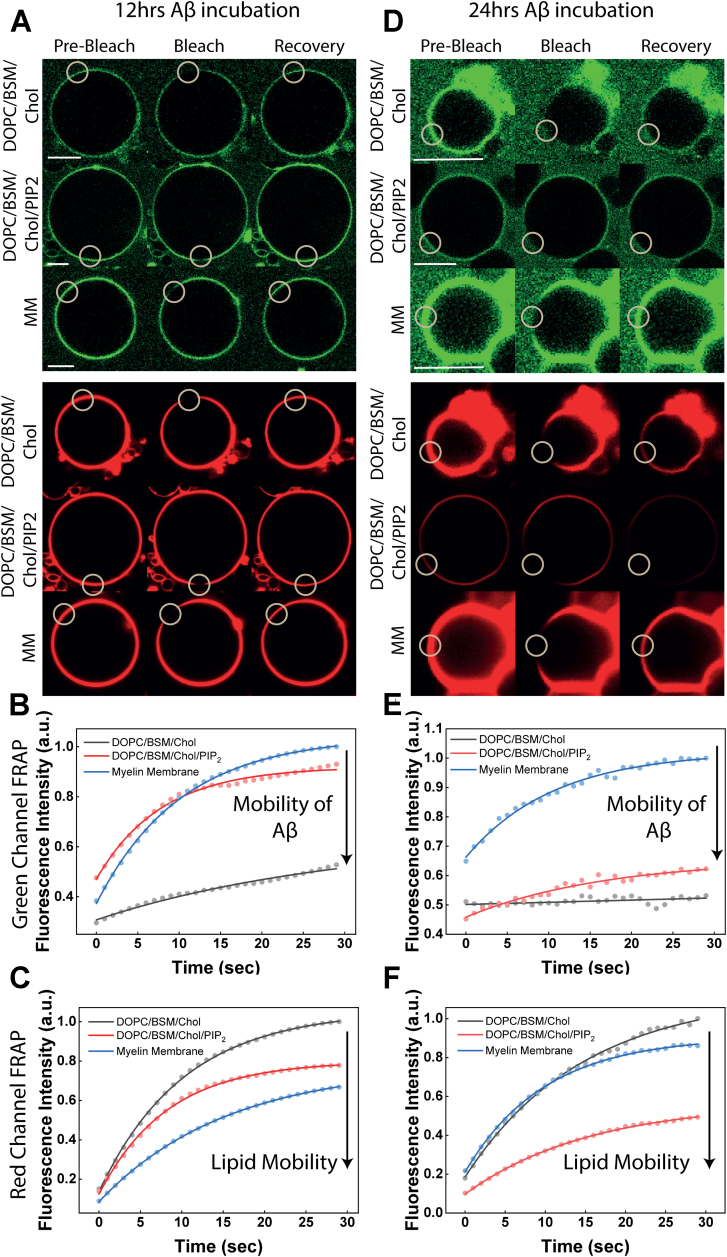
**Changes in the diffusion of Aβ-40 and lipid membrane during aggregation.***A*, representative fluorescence recovery after photobleaching (FRAP) images of the *green channel* (Aβ-40 doped with Hylite-488 Aβ-40) and the *red channel* (lipid membrane doped with rhodamine PE) showing the Pre-bleach, bleach, and Recovery of the three conditions (DOPC/BSM/Chol (4:4:2), DOPC/BSM/Chol/PIP_2_ (2:4:3:1) and the myelin-like model) marked with a *white circle* to denote the region of interest in each case at the 12 h time point. *B*, normalized fluorescence recovery curves after photobleaching for Aβ-40 bound (*green channel* - peptide) to the above-mentioned three membrane conditions at the 12-h time point. *C*, normalized fluorescence recovery curves after photobleaching for Aβ-40 bound (*red channel* – lipid membrane) to the above-mentioned three membrane conditions at the 12-h time point. *D*, representative fluorescence recovery after photobleaching (FRAP) images of the green channel (Aβ-40 doped with Hylite-488 Aβ-40) showing the Pre-bleach, bleach, and Recovery of the three conditions (DOPC/BSM/Chol (4:4:2), DOPC/BSM/Chol/PIP_2_ (2:4:3:1) and the myelin-like model) marked with a *white circle* to denote the region of interest in each case at the 24 h time point. *E*, normalized fluorescence recovery curves after photobleaching for Aβ-40 bound (*green channel* - peptide) to the above-mentioned three membrane conditions at the 24-h time point. *F*, normalized fluorescence recovery curves after photobleaching for Aβ-40 bound (*red channel* – lipid membrane) to the above-mentioned three membrane conditions at the 24-h time point. All FRAP curves in *black lines* are for DOPC/BSM/Chol (4:4:2), *red lines* are for DOPC/BSM/Chol/PIP_2_ (2:4:3:1), and *blue lines* represent curves for the myelin-like model membrane. Each curve is a mean of 5 independent experiments. The scale bar for FRAP images is 10 μm. BSM, sphingomyelin (Brain, Porcine); DOPC, 1,2-Dioleoyl-sn-glycero-3-phosphocholine; DOPS, 1,2-dioleoyl-sn-glycero-3-phospho-L-serine; DPPC, 1,2-dipalmitoyl-sn-glycero-3-phosphocholine; GUV, giant unilamellar vesicles; PIP2, L-α-phosphatidylinositol-4,5-bisphosphate

Another indication of the nature of the binding of Aβ-40 and the effect on the membrane lipid diffusion can be drawn from the fluorescence recovery after photobleaching (FRAP) curves of fluorescently labeled Aβ-40 bound to the membrane at different time points. FRAP curves would suggest if the membrane-bound Aβ-40 is in a rigid immobile or a mobile structural arrangement as well as the consequent effect on the membrane lipid diffusion. We, therefore, investigated the fluorescence recovery of Aβ-40 on membrane conditions as chosen earlier, at 12 h and 24 h. The reason for selecting the mid to late phase of fibrillation (*i.e.*, 12 and 24-h time points) is based on the observation that the predominant pool of GUVs show comparable binding at 12 and 24 h and therefore allow bleaching of a region of interest (ROI) on the GUV equatorial plain (Fig. 6, Table S2, and Movies S1–S6). We observed complete recovery of fluorescent signal from Aβ-40 for the myelin-like model membrane (containing the highest lipid packing defects) at 12 h, suggesting a dynamic interaction of Aβ-40 at the membrane interface. Photobleaching at 24 h could only bleach 35 to 40% of the fluorescence at the ROI, likely, due to a dense coating of Aβ-40 fibrils on the membrane, which was recovered fully (Fig. 6, *A*, *B*, *D* and *E*). Likewise, ∼90% of the Aβ-40 fluorescence was found to recover upon photobleaching of the membrane containing moderate defects (DOPC/BSM/Chol/PIP2) at 12 h, which eventually decreased by one-third at 24 h (Fig. 6, *A*, *B*, *D* and *E*). No significant fluorescence recovery of Aβ-40 was observed in the case of the membrane with the least defects (PC/SM/Chol) both at 12 and 24 h (Fig. 6, *A*, *B*, *D* and *E*). Aβ-40 seems to be in a highly dynamic interaction with the myelin-like model membrane resulting in significant extraction of lipid tubules. This, in turn, leads to a generation of free space on the GUVs, allowing continuous binding as evident from the recovery of the Aβ-40 signal at 24 h. The enhanced fluidity of the myelin, in turn, helps amyloid aggregation (33). Indeed, monitoring the FRAP curves of the myelin membrane lipids suggest a ∼20% increased recovery in fluorescence at 24 h compared to that at 12 h, after photobleaching (Fig. 6, *C* and *F*). A twofold drop in the fluorescence recovery in the lipid channel at 24 h compared to 12 h, in the case of DOPC/BSM/Chol/PIP2 membrane, suggests a restricted movement of the lipids and more stable interfacial interaction (Fig. 6, *C* and *F*). Finally, in the case of the DOPC/BSM/Chol (membrane with the least amounts of defects), no significant change in the fluorescence recovery signal in the lipid channel is observed at 12 and 24 h (Fig. 6, *C* and *F*).

### Aβ-40–mediated changes in phase behavior and compressibility modulus of membrane monolayer at a short timescale

**Figure 7:**
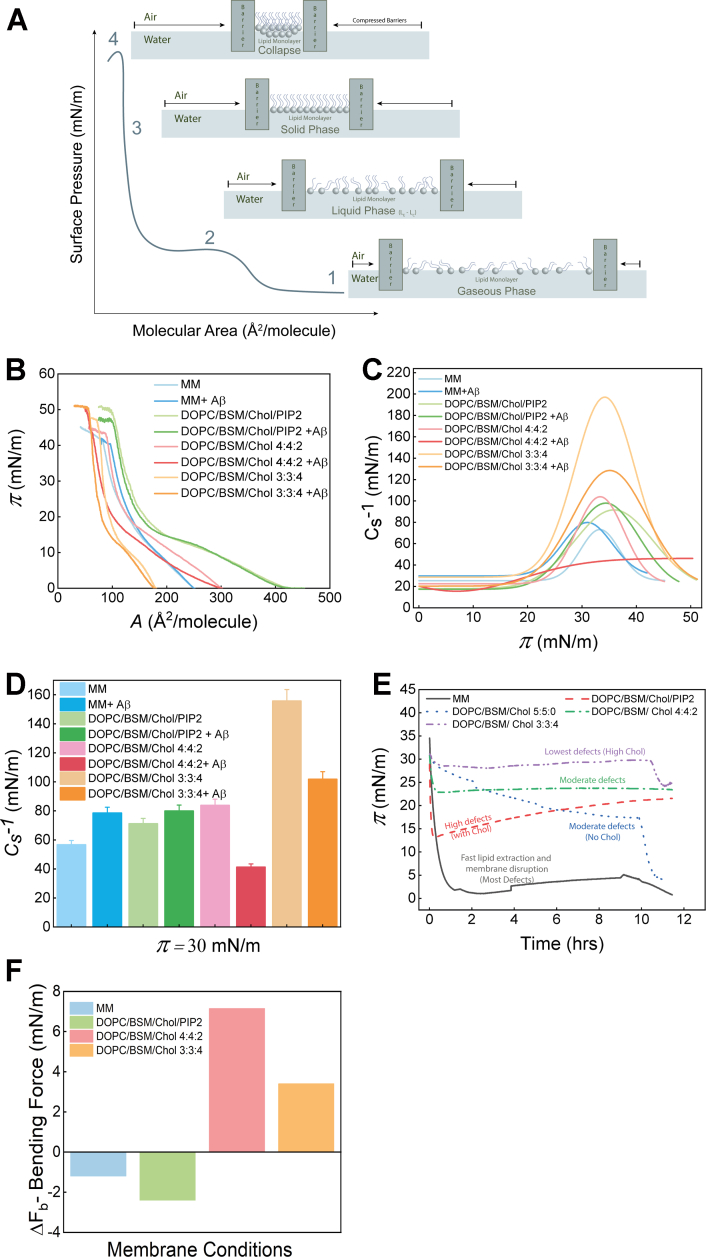
**Phase behavior, compressibility modulus, and load generation induced by Aβ-40.***A*, illustration of typical surface pressure (*π*)– mean molecular area (*A*) isotherm with a visual representation of the molecular alignment of the lipid molecules at different phases of the isotherm. *B*, *π - A* isotherm for the different membrane conditions that include myelin-like model membrane with and without Aβ-40 (*blue* and *light blue*), DOPC/BSM/Chol/PIP2 (2:4:3:1) membrane with and without Aβ-40 (*green* and *light green*), DOPC/BSM/Chol (4:4:2) membrane with and without Aβ-40 (*pink* and *light pink*) DOPC/BSM/Chol (3:3:4) membrane with and without Aβ-40 (*orange* and *beige*) at 25 °C. *C*, compressibility modulus (*C*_*s*_^*−1*^) *–* surface pressure (*π*) curves for each control monolayer model as well as in the presence of Aβ-40 are shown in the graph following the same labeling order and color scheme for the membrane conditions as above. *D*, compressibility moduli (*C*_*s*_^*−1*^) at a surface pressure of 30 mN/m (at the bilayer equivalence pressure) is shown in the graph with the same labeling order and color scheme for the membrane conditions as above. *E*, surface pressure (*π*)- Time plot for different membrane conditions that include myelin-like model (*black line*), DOPC/BSM/Chol/PIP2 (2:4:3:1) (*red line*), DOPC/BSM/Chol (5:5:0) (*blue line*), DOPC/BSM/Chol (4:4:2) (*green line*), DOPC/BSM/Chol (3:3:4) (*purple line*) at 25 °C for 12 h. *F*, Bending force (ΔF_b_), a measure of deformability, of different membrane conditions is shown in the histogram. BSM, sphingomyelin (Brain, Porcine); DOPC, 1,2-Dioleoyl-sn-glycero-3-phosphocholine; DOPS, 1,2-dioleoyl-sn-glycero-3-phospho-L-serine; DPPC, 1,2-dipalmitoyl-sn-glycero-3-phosphocholine; GUV, giant unilamellar vesicles; PIP2, L-α-phosphatidylinositol-4,5-bisphosphate.

Bilayer experiments allowed us to probe the long time-scale phenomena (*i.e.*, from 1 h to 24 h) that cannot capture the molecular aspects of interaction during the earliest time scales. We therefore next aimed to investigate the short time-scale phenomena capturing the molecular events of the earliest binding as well as changes in the mechanical properties of the membrane within 1 h. To address this, we used two-dimensional models of a biological membrane, that is, Langmuir monolayers which are highly sensitive tools to study mixing behavior, and binding/insertion (34, 35, 36). The surface pressure-Area (*π-A*) isotherms capture the modulation of the phase behavior and collapse pressure of the membrane induced by Aβ-40 interaction upon compression of a free-standing monolayer, particularly in the early time scales (within 1 h). We observed that the *π-A* isotherm of the myelin-like model membrane in the presence of Aβ-40 shifts towards the right starting at a surface pressure of 15 mN/m, indicating an increase in the area per molecule in comparison to the control myelin monolayer isotherms. This led us to conclude that Aβ-40 tends to get incorporated in the monolayer, which justifies the shift driven by the expulsion of some lipids out of the monolayer observed by the differences in the monolayer collapse pressure (*blue* and *light blue isotherms*, Fig. 7, *A* and *B*). An interesting difference in the behavior of the DOPC/BSM/Chol/PIP2 membrane in the presence and absence of Aβ-40 (*green* and *light green isotherms*) is noteworthy. Although both the isotherms appear similar until the collapse pressure is approached, however, a distinct plateau is observed indicative of the coexistence of a liquid-expanded to liquid-condensed (L_e_-L_c_) region. A slight shift towards the left is observed at the coexistence plateau, indicating a decrease in the area per molecule. This observation hints at squeezing out of the lipids from the monolayer as a result of the crowding on the head groups due to the condensing effect of Aβ-40 interaction with the lipid monolayer. This is further corroborated by the difference in the collapse pressure indicative of lipid loss during the compression.

We then looked at the mixing behavior of the DOPC/BSM/Chol (4:4:2) membrane and Aβ-40. A left shift quite early in the isotherm was observed seen just at the start of the L_e_ phase indicating Aβ-40 induced condensation upon compression. The collapse of the membrane condition with Aβ-40 in the subphase happens to be higher (∼50 mN/m) than the control without Aβ-40. This condensing effect caused by Aβ-40 stabilized the solid phase to a greater extent indicated by an increase in the surface pressure corresponding to the collapse of the membrane models. The higher surface pressure collapse of these membranes could also be attributed to Aβ-40 having a lesser degree of affinity to these membranes, albeit having enough affinity to non-disruptively condense and stabilize the membrane. A similar observation was found for a membrane containing DOPC/BSM/Chol in 3:3:4.

We, further, quantified the changes in the elastic compressibility modulus (*C*_*s*_^*−1*^) of the different membranes induced by the Aβ-40 interaction as described earlier (See Experimental procedures for details). This parameter reports the in-plane elasticity (compressibility modulus) of the monolayer films and is also correlated with the different phases of the compression isotherms, namely, Gaseous (G), Liquid expanded (LE), Liquid condensed (LC), and Solid (S). *C*_*s*_^*−1*^ of 12 mN/m to 100 mN/m corresponds to the LE region, *C*_*s*_^*−1*^ ranging from 100 to 250 mN/m corresponds to LC, and *C*_*s*_^*−1*^ of 250 mN/m and above corresponds to S (37). Particularly, the *C*_*s*_^*−1*^ of a given membrane monolayer at surface pressure (π) of 25 to 30 mN/m is considered to reflect the elasticity of a bilayer membrane as the lateral pressure and mechanical properties of monolayer are known to be similar to that of a bilayer at this pressure (38, 39). The *C*_*s*_^*−1*^ of the myelin-like model membrane was close to ∼70 mN/m, which reaches a value above ∼80 mN/m upon the interaction of Aβ-40. Similarly, in the case of the DOPC/BSM/Chol/PIP2 membrane, the *C*_*s*_^*−1*^ was found to be ∼ 85 mN/m, which slightly increased to ∼100 mN/m upon Aβ-40 interaction.

Together, the observed slight increase in the *C*_*s*_^*−1*^ suggests that the interaction of Aβ-40 with the above two membrane conditions decreases the elastic behavior of the membranes, evident from the observation that the two membrane conditions collapse within the LE phase.

Interestingly, in the case of the DOPC/BSM/Chol (4:4:2) membrane, the *C*_*s*_^*−1*^ peaked at the value bordering ∼100 mN/m that decreased to around ∼50 mN/m upon the interaction of Aβ-40. This means that on interaction with Aβ-40, the membrane becomes more compressible. The DOPC/BSM/Chol (3:3:4) membrane was found to be the most inelastic of all the membrane compositions used, reaching a *C*_*s*_^*−1*^ above ∼200 mN/m, that dropped to ∼130 mN/m upon the interaction of Aβ-40. It is noteworthy that both the membrane conditions showed a drastic decrease in their *C*_*s*_^*−1*^, which translates into more elastic behavior of the membranes. Thus, analyzing the trends of the *C*_*s*_^*−1*^ values for each condition at 30 mN/m gives a biologically closer understanding of the changes in the elastic behavior of the membrane induced by interacting Aβ-40 that showed the same trend as observed for the peak values of *C*_*s*_^*−1*^ (Fig. 7, *C* and *D*). The base compressibility modulus of the myelin-like model membrane was ∼56 mN/m which increases to ∼78 mN/m in the presence of Aβ-40, which translates to approximately 38% increase in compressibility modulus of the model or, in other words, a decrease in the monolayer compressibility. The DOPC/BSM/Chol/PIP2 membrane condition shows a similar trend where the base compressibility modulus is observed to be ∼71 mN/m and increases to ∼80 mN/m, which is a 12% increase in the compressibility modulus. Remarkably, these changes in membrane compositions that were relatively less complex and devoid of essentially PI showed a reverse trend. While in the case of DOPC/BSM/Chol 4:4:2 membrane condition, the base compressibility modulus was observed to be ∼84 mN/m, which drastically decreased to approximately half of it, that is, ∼41 mN/m on incubating it with Aβ-40. A ∼50% decrease in compressibility modulus, which in simpler terms means a drastic increase in the monolayer compressibility. A similar trend in the DOPC/BSM/Chol 3:3:4 membrane condition is also observed where base compressibility modulus is ∼155 mN/m, and after incubation with Aβ-40 drops to ∼101 mN/m, which is a ∼30% decrease in the compressibility modulus.

### Aβ-40 fibril load generation on monolayers compressed at bilayer lateral pressure

After establishing the mixing behavior of Aβ-40 with free-standing monolayer and its effect on elasticity, we next wondered how would Aβ-40 binding/fibrillation affect lipid monolayer membranes compressed to a surface pressure of 30 to 35 mN/m to mimic bilayer lateral pressure throughout fibrillation. The membrane monolayer was allowed to equilibrate for 15 to 20 min, after which the Aβ-40 was injected into the monolayer sub-phase (Fig. 7*E*). It was observed that immediately after injection of Aβ-40 within 1 h, there was a drastic drop in the surface pressure of the myelin-like model membrane from ∼30 mN/m to ∼2 mN/m (Fig. 7*E*). This indicates that the peptide was inducing aggregation-based expulsion of the lipids into the subphase as it tried to populate the air/water interface already crowded by the lipid monolayer and, in the process, disrupting the membrane. Similarly, this was also observed in the case of DOPC/BSM/Chol/PIP2 but to a lesser degree, where the drop was about ∼16 mN/m. In the case of both DOPC/BSM/Chol 4:4:2 and 3:3:4, there was a drop of ∼7 mN/m and ∼2 mN/m. Interestingly, the observed behavior of surface-pressure drop recapitulated our previous observations, wherein, Aβ-40–induced membrane disruption followed a similar order, that is, myelin-like model membrane > DOPC/BSM/Chol/PIP2 > DOPC/BSM/Chol (4:4:2) > DOPC/BSM > DOPC/BSM/Chol (3:3:4). We reasoned that this could be because of the number of lipid packing defects available for Aβ-40 to embed itself at the interface. The much faster disruption of the membrane monolayers in comparison to the visible deformation in GUVs could be attributed to the lack of *trans*-bilayer interdigitation or leaflet coupling as well as the high sensitivity to the surface pressure changes in monolayer experiments.

## Discussion

In this study, we first showed that the binding of Aβ-40 to the reconstituted myelin-like model membrane, although homogenous, shows an oscillating pattern of binding intensity at early (1–4 h), mid (4–12 h), and late phase (12–24 h) (Fig. 1). The differences in binding might arise due the existence of a heterogeneous population of Aβ-40 that results in different modes of interactions (40). It is also likely to be influenced by the changes in the membrane elasticity properties triggered by the preceding pool of the bound Aβ that might dissociate as the membrane property changes. The binding of several classes of polymerizing proteins is known to be sensitive to the membrane elasticity parameters (41, 42). We then show that extensive tubulation of the myelin-like model membrane is observed in the late phase involving lipid sequestration by the growing fibril (Fig. 1). Our work captures visual evidence of the fibril-induced lipid extraction *in vitro* and in line with the growing body of evidence that suggests the presence of lipids in mature fibrils or plaques *in vivo* (7). Indeed, recent clinical evidence suggests myelin lipid loss is one of the early pathological features for the progression of AD (1, 43). Although the reconstituted myelin-like model membrane enhances sustained aggregation of Aβ-40, however, lipid specificity of Aβ-40 revealed that except PIP2 other myelin lipid components such as DOPC, DOPG, PI, and DOPS decrease the overall aggregation rate (Fig. 2*D*). The delayed binding to DOPC membranes (Fig. 4*A*) is in line with previous observations that Aβ monomers do not directly interact with the DOPC membrane till fibrils are formed (44). While significant binding of Aβ-40 to the DOPG membrane was observed, however, it did not affect the aggregation kinetics (Figs. 2*A* and 4, *D* and *E*) in line with previous observations (35). The absence of significant binding of Aβ-40 to DOPS membranes can be attributed to the absence of Ca^2+^, essential for bridging Aβ nucleation on the membrane (45). DOPE and PI membranes were also found to retard the Aβ-40 aggregation in line with previous observations that suggested PE membranes hamper fibril formation and that PI can help fibril elongation but not nucleation (46, 47). Indeed, different constituent lipids of myelin membrane, that differ in their geometry and net charge, might trigger lipid-induced depolymerization of Aβ fibrils generating “reverse oligomers” having different membrane binding propensity (48, 49, 50). The observed modulation of the Aβ-40 binding and fibrillation by constituent lipids of the myelin membrane highlights the importance of lipid specificity in membrane remodeling during aggregation.

The diverse geometry of lipids in myelin membrane and their entropically driven propensity to readily mix should result in the highest lipid packing defect density compared to membranes with less heterogeneous lipid shapes (51, 52), as is also quantified computationally (Fig. 3). Among the membrane conditions chosen (with varying lipid defect densities and charge) to investigate the mechanism, the general trend for the average binding intensity of Aβ-40 during the early, mid, and late phases is myelin-like model membrane > PC/BSM/Chol/PIP2 > PC/BSM/Chol that reflect the decreasing density of the lipid packing defects at the membrane interface (Figs. 1 and 3). DOPC although conical in shape yet lacks of electrostatics forces resulting in negligible to weak binding of Aβ-40 (Fig. 4*A*). In addition to the lipid packing defects, net negative charge and the presence of cholesterol might also facilitate the observed binding (53, 54). The Aβ-40–induced membrane deformation resulting in lipid association with fibril progresses through the membrane fluidization (Figure 3, Figure 4, Figure 5). We cannot rule out the possibility that a change in the anisotropy of the membrane may also result from Aβ-40 binding without deformation. However, enhanced fluidity makes the membrane more deformable, thus explaining the observed lipid/fibril tubular structures. Indeed, Aβ-40 was reported to fluidize the neuronal membrane in a cholesterol-dependent manner (55, 56). We then show that the observed changes in the fluidity of the membrane also reflect in the diffusion of both Aβ-40 and lipids. The diffusion of Aβ-40 bound to the myelin-like model membrane did not undergo any significant change at the observed time regimes suggesting highly dynamic interaction (Fig 6, *B* and *E*). Interestingly, lipid diffusion in myelin-like model membrane increased reasonably over time suggesting fluidization of the membrane (Fig 6, *C* and *F* and Table S1). The observed lack of change or reduction in lipid diffusion in membranes with lower defect densities (Fig. 6) could be attributed to the creation of Aβ-40–induced diffusion barriers (16, 57). This further reinforces the idea that lipid packing defect densities and charge are important in driving dynamics of Aβ-40 interaction resulting in lipid extraction with growing fibril.

**Figure 8:**
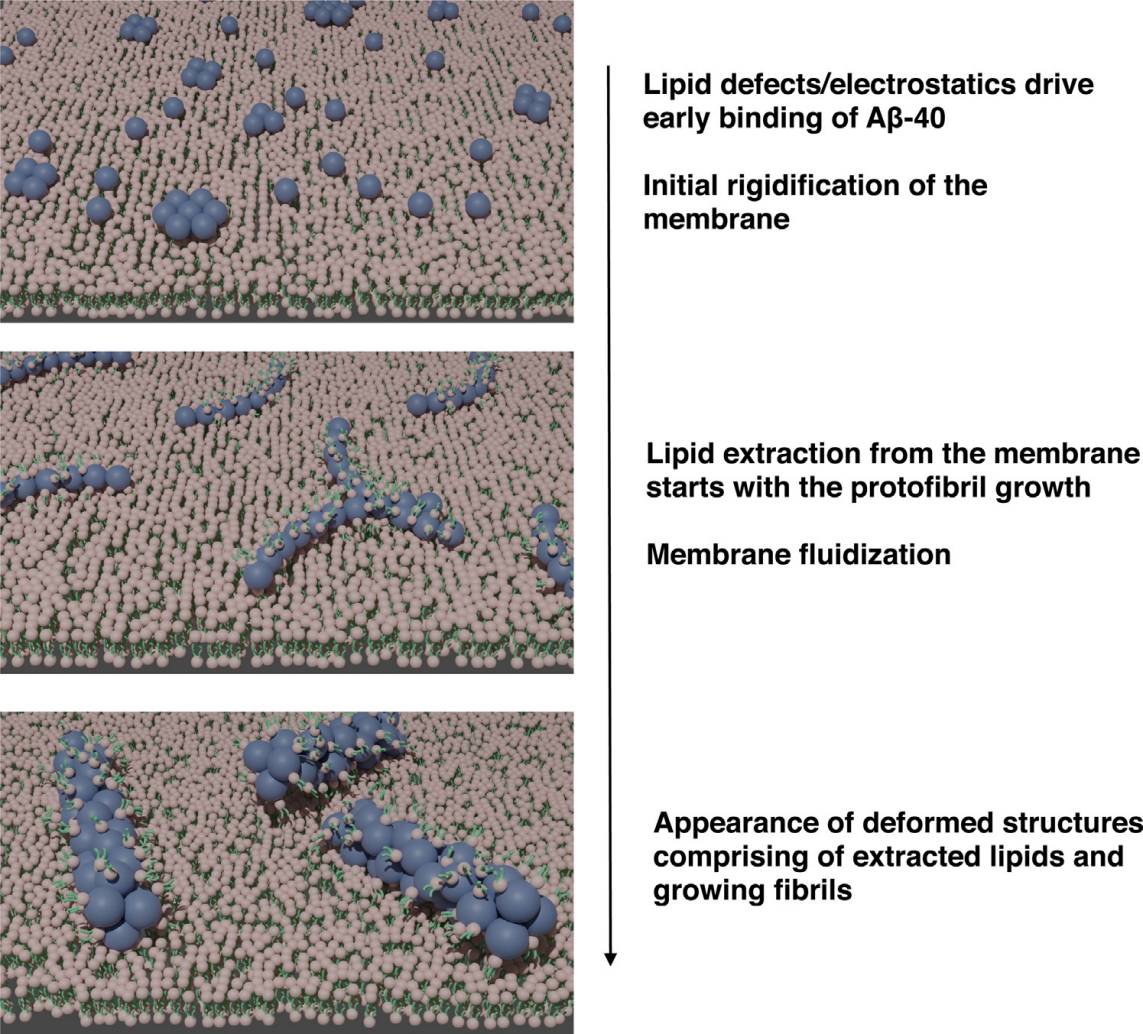
**A schematic of the proposed model.** A schematic of the proposed model for the progression of the neuronal myelin membrane deformation mediated by Aβ-40 aggregation.

Although Aβ-40 mediated fluidization of the myelin-like model membrane precedes the generation of lipid/fibril tubular structures, the binding of Aβ-40 increases the compressibility modulus (*C*_*s*_^*−1*^) of the model membrane within 30 min of binding, surprisingly, suggesting rigidification of the membrane. This could likely be due to the binding of the peptide to the membrane defect sites (Fig. 7*A*) (58). On the contrary, Aβ-40 was found to fluidize the membranes with relatively less lipid packing defect densities and electrostatic factors (*i.e.*, ternary mixtures with varying PC/SM/Cholesterol) right from the beginning (Fig. 7*C*). The striking reduction in the surface pressure of the myelin-like monolayer equilibrated with Aβ-40 within 1 h can be attributed to load generated by the growing oligomeric/protofibrils resulting in the extraction of lipids and the lack of interleaflet coupling in the membrane monolayer (59, 60). Recently, the insertion of oligomers and protofibrils on liposomes was also visualized using 3D Cryo-electron tomography (13). We think that although the monomeric or low oligomeric Aβ-40 may insert into the membrane lipid packing defect, however, the fibrillation takes place at the membrane interface as evident from the lack of increase in surface pressure corresponding to surface insertion (Fig. 7). Together, the work captures molecular insights of both early and late events that result in progression of the Aβ-40–mediated myelin membrane deformation. The findings demonstrate how lipid packing defect density and electrostatic interactions drive the binding of Aβ-40 and the role of consequent manipulation of the fluidity, diffusion, and compressibility modulus of the myelin membrane in inducing lipid extraction by the growing fibril as proposed in the schematic model (Fig. 8) (7). The modulation of lipid packing and protein recruitment by changing the lipid composition has been shown important for several peripheral proteins (51). More generally, the work also provides mechanistic insights into the transition between early pore formation and late fibril-mediated lipid extraction that contributes to the two-step mechanism (14, 61, 62, 63). Any correlation between the kind of lipid and fibril morphology remains to be explored and could be of interest for pathological significance.

## Experimental procedures

1,2-Dioleoyl-sn-glycero-3-phosphocholine (DOPC), 1,2-dioleoyl- sn-glycero-3-phosphoethanolamine (DOPE), L-α-phosphatidylinositol (liver PI), 1,2-dipalmitoyl-sn-glycero-3-phosphocholine (DPPC), 1,2-dioleoyl-sn-glycero-3-phospho-L-serine (DOPS), DOPG, PIP2, BSM, L-α-phosphatidylinositol-4,5-bisphosphate (Brain, Porcine), 1,2-dioleoyl-sn-glycero-3-phosphoethanolamine-N-(lissamine rhodamine B sulfonyl) (Rhod PE), and cholesterol were purchased from (Avanti Polar Lipids). Composition of myelin-like model membrane—DOPC/BSM/DOPE/PI/DOPS (4:3:1:1:0.4) supplemented with 0.6 mol Cholesterol/mol (phospholipid). Thus, the total percentage of cholesterol amounts to ∼60% compared to the total phospholipid in the chosen composition. Rhod PE is known to preferentially partition into liquid-disordered phases and is thus used to visualize the same (64). Beta-Amyloid (1–40) (DAEFRHDSGYEVHHQKLVFFAEDV-GSNKGAIIGLMVGGVV), HiLyte Fluor 488 – labeled Beta – Amyloid (1–40) (HiLyte Fluor 488-DAEFRHDSGYEVHHQKLVFFAEDVGSNKGAIIGLMVGGVV), Human, Anaspec, TMA-DPH from Invitrogen Thermo Fisher Scientific was used for the fluorimetry experiments.

### Peptide reconstitution

The commercially available amyloid Beta 40 (Aβ 40) was purchased from AnaSpec, Inc (purity ≥ 95%) which was stored at −20 °C. At the time of preparation, the stored peptide was made to equilibrate at room temperature. The peptide powder was then dissolved in 40 μl of 1% NH_4_OH diluting it with Milli-Q water up to 1 ml, bringing the concentration of the peptide to 1 mg/ml. Further, 10 μl aliquots of this preparation were flash frozen and lyophilized, which was then stored at −20 °C. The peptide was then dissolved in the desired buffer for further experiments. The reconstituted peptide was incubated for 2 h to allow aggregation before quantifying different populations of soluble forms of Aβ-40 by fluorescence correlation spectroscopy.

### Thioflavin T assay for the measurement of fibrillation kinetics of Aβ 40

The freeze-dried peptide was then reconstituted in PBS. The Final concentration of the peptide for Thioflavin T (ThT) experiments was kept at 1 μM. The ThT concentration used for the experiments was 20 μM. The lipid specificity of Aβ 40 was screened by incubating the peptide with giant GUVs which acted like lipid templates for amyloid aggregation. The fluorescence intensity was followed against time to monitor the Aβ 40 fibrillation kinetics using BioTek Synergy H1 fluorescence plate reader at an excitation wavelength of 440 nm and an emission wavelength of 490 nm. Readings in triplicate were recorded every 30 min for 6 h. To minimize evaporation, an Opti-seal was applied over the microplate. The data were then normalized by the lipid controls for each condition and plotted using Origin pro. The initial growth rate was calculated by fitting the initial log phase of the aggregation kinetics (ThT fluorescence assay) to the equation y = A + B∗exp(−kx).

### Preparation of large unilamellar vesicles

For each membrane condition, 1 mM stock solution of the required lipid was prepared and dried under a gentle nitrogen gas stream; subsequently, it was vacuum dried for an hour to remove the residual solvent from the lipid film. These lipid films were then rehydrated in 1 ml of PBS of pH 7.4 and then were incubated for about 15 min in a water bath, making sure the temperature remained above the transition temperature of the lipid. The heated samples were then vortexed for 4 to 5 min. For the preparation of large unilamellar vesicles (LUVs), the MLV suspension was then sonicated for 5 min at 0.9 pulse rate and 100% amplitude. The size of the LUVs was confirmed using DLS with the average diameter of the LUVs being ∼150 nm.

### Preparation of fluorescently labeled GUVs

The gel-assisted described by Weinberger *et al.* (65) was followed for the preparation of GUVs. Briefly, a 5%(w/w) solution of polyvinyl alcohol (PVA) was prepared in deionized water, 300 μl of this solution was evenly spread on a plastic Petri dish and dried at 50 °C for 30 min in an oven. From a solution of lipids in chloroform at 1 mg ml^−1^ concentration doped with 1 mol% rhodamine-PE, 20 μl of this solution was spread on the PVA-coated Petri dish. These Petri dishes were then placed under vacuum for 45 min to sufficiently dry the lipid film. To prevent dewetting, the Petri dishes were cleaned with UV for 15 min. This lipid film was then left to swell in PBS at a pH of 7.4 for 45 min. The hydrated vesicles were gently dislodged and transferred to a microcentrifuge tube using a pipette (66).

### Confocal fluorescence microscopy

A custom-made chamber was used for incubating the GUVs and Aβ 40 doped with 10% Hylite-488 Aβ 40. The coverslip was wiped cleaned with 70% ethanol and was then air-dried. Then, 90 μl of the GUVs from the microcentrifuge tube was added to the chamber along with 10 μl of Aβ 40 in equi-osmolar PBS buffer with the effective concentration of the peptide being 2.5 μM. This chamber was then sealed with an opti-seal to minimize evaporation during prolonged incubation of the sample. Imaging was performed on a Leica TCS-SP8 confocal instrument using appropriate lasers for rhodamine-PE (DPSS-561) and Hylite-488 Aβ 40 (argon-488). Identical laser power and gain settings were used during all the experiments. The image processing was done using ImageJ.

### Quantification of membrane packing defects

The biological membranes are composed of lipids of different shapes such as cylindrical, conical, etc., and when such lipids get together to frame a bilayer structure with their polar heads exposed to the aqueous environment, several voids get created which leads to the packing defects in the membrane. To study these defects, membranes of four different compositions were set up and simulated using coarse-grained (CG) molecular dynamics. The CG simulations were performed using the MARTINI version 2.2 (67) force field in GROMACS version 5.1 (68). The four membrane systems with the varying compositions are: (1) pure DOPC, (2) DOPC:BSM:CHOL at a molar ratio of 4:4:2, (3) DOPC:BSM:CHOL:PIP2 at a molar ratio of 2:4:3:1, (4) myelin-like model membrane: DOPC/BSM/DOPE/PI/DOPS(4:3:1:1:0.4) supplemented with 0.6 mol Cholesterol/mol (phospholipid). The python script, insane.py, was used to generate each of the above-mentioned membrane systems within a simulation box of 12 × 12 × 10 nm^3^ solvated with explicit MARTINI water and with appropriate numbers of Na+ and Cl^−^ counter ions to make the charge of each of the system neutral (43). After running the steepest descent minimization, a 200 ns NVT equilibration was executed with the temperature coupling by velocity rescaling the thermostat to a reference temperature of 300 K. The NVT equilibration is followed by a 200 ns NPT equilibration with the pressure coupling by Parinello-Rahman barostat to a reference pressure of 1 bar. After the equilibration, a 10 μs production run for each of the bilayer systems was performed out of which the last 1 μs was used for analysis.

We used PackMem (28) to quantify the lipid packing defects. PackMem follows the Cartesian grid system for mapping of the membrane surface where the grid dimension is set to 1 Å × 1 Å. This tool computes the defects by characterizing them into deep and shallow defects. The deep defects represent the voids created due to the presence of aliphatic atoms deeper than d Å (where the value of d is set to 1 Å) below the central atom of glycerol whereas shallow defects represent the accessible aliphatic atoms that are less than d Å below the central atom of glycerol and all types represent the combination of both the deep as well as shallow defects (2). For the execution of PackMem, the MARTINI trajectory files obtained from the production run were taken as input to generate the pdb frames saved every 200 ps. The script “ScriptPackMem.sh” then takes each pdb file as input to calculate deep and shallow defects. The distribution of packing defects area follows a mono-exponential decay,

(1) $$P\left(A\right)=bexp\left(\frac{-A}{\pi }\right)$$

where *P(A)* denotes the probability of finding a defect with area *A*, *b* is the pre-exponential factor and *π* is the packing defect constant (28). Finally, an R script provided with the package computes the mean packing defect constants. The barplot for the packing defect constants for each type of packing defect is prepared using GNUPLOT version 5.2 (69).

### Fluorescence recovery after photobleaching

For the Fluorescence recovery after photobleaching (FRAP) measurements on the GUVs, the GUVs were doped with 1 mol% of rhodamine PE and were incubated with 2.5 μM of Aβ 40 doped with 10% Hylite-488 Aβ 40. First, pre-bleach images at an attenuated laser intensity were acquired. Photobleaching was performed using DPSS-561 (to photobleach the lipid rhodamine-PE) and argon-488 (to photo-bleach bound the bound Hylite-488 labeled Aβ 40) at 100% laser power for 30 s, achieving a partial bleach through a circular ROI of a nominal radius r = 2.2 to 2.4 μm. The laser was then switched back to the attenuated intensity, and the recovery curve along with the images was recorded for several seconds. The photobleaching was executed at the equatorial plane of the GUV being visualized. The FRAP curves for each condition were repeated five times and then normalized. The diffusion coefficient was calculated using the Soumpasis equation for 2D-diffusion

(2) $$D_{r}=0.224\frac{r^{2}}{\tau_{1/2}}$$

where 0.244 is the numerically determined value, r (2.2 μm) stands for the radius of the laser beam focused on the region of interest, and τ_1/2_ is the time required for half the recovery. The time for half the recovery was determined by plotting the normalized recovery curve.

### Fluorescence spectrophotometric assay

The Fluorescence spectroscopy experiments were performed on LUVs (preparation described earlier). The TMA-DPH was dissolved in DMSO to prepare a final concentration of 2 mM. The LUVs prepared were then incubated with Aβ 40 at an effective concentration of 1 μM in presence of 1 μM TMA-DPH and made up the total volume of the LUV including the Aβ 40 and TMA-DPH to 1 ml. This mix was then incubated in dark. The fluorescence anisotropy was measured in Photoluminescence Spectrometer FLS 1000 for different time points, that is, fourth and eighth hour by exciting at 360 nm and emission at 430 nm. A control experiment was carried out without peptide. The 0.1% (V/V) of DMSO is known not to affect the spectroscopic data. The anisotropy (r) was automatically calculated by the instrument using the equation:

(3) $$r=\frac{I_{VV}-{GI}_{VH}}{I_{VV}+{2GI}_{VH}}$$

where I_VV_ and I_VH_ are the measured fluorescence intensities with the excitation polarizer oriented vertically and the emission polarizer oriented vertically and horizontally, respectively. G (=I_HV_/I_HH_) is the grating correction factor that corrects for wavelength-dependent distortion of the polarizer. All experiments were conducted with multiple sets of samples.

### Fluorescence correlation spectroscopy experiments and data analysis

Fluorescence correlation spectroscopy experiments were carried out using a dual-channel ISS Alba V system equipped with a 60× water-immersion objective (NA 1.2). Samples were excited with an argon laser at 488 nm. All protein data were normalized using the *τ*_*D*_ value obtained with the free dye (Alexa488) which was measured under identical conditions. For a single-component system, the diffusion time (*τ*_*D*_) of a fluorophore and the average number of particles (*N*) in the observation volume can be calculated by fitting the correlation function [*G(τ)*] to Equation 1:

(4) $$G\left(\tau \right)=1+\left(\frac{1}{N(1+\frac{\tau }{\tau_{D}}}\right)\;\frac{1}{\sqrt{1+S^{2}\frac{\tau }{\tau_{D}}}}$$

where, *S* is the structure parameter, which is the depth-to-diameter ratio. The characteristic diffusion coefficient (*D*) of the molecule can be calculated from *τ*_*D*_ using Equation 2:

(5) $$\tau_{D}=\frac{\omega^{2}}{4D}$$

where, *ω* is the radius of the observation volume, which can be obtained by measuring the *τ*_*D*_ of a fluorophore with a known *D* value. The value of the hydrodynamic radius (*r*_*H*_) of a labeled molecule can be calculated from *D* using the Stokes-Einstein equation [Equation 6]:

(6) $$D=\frac{\kappa T}{6\pi \eta r_{H}}$$

where *k* is the Boltzmann constant, *T* is the temperature and *η* corresponds to the viscosity of the solution (70).

### Transmission electron microscopy

100 μl of 200 μM LUV solution was incubated with 1 μM Aβ 40 for different chosen time points. 10 μl of the incubated sample was then added to a carbon-coated copper grid and this was left for 2 min, it was later wicked off with filter paper. The grid was then rinsed with deionized water and a 5 μl 4% uranyl acetate replacement (EMS) droplet was placed onto the grid. After minutes, this solution was wicked off and the grid was air-dried. The imaging was performed on a JEOL (JEM 2100F) microscope with an operating voltage of 200 kV.

### Monolayer experiments

Langmuir monolayer films were spread on a Teflon molded trough (Apex Instruments, India) having an inner working dimension of 305 mm × 105 mm. The rectangular trough was equipped with two movable Teflon barriers that provide symmetrical compression. The system is equipped with an electronic balance having a sensitivity of ±0.5 mN/m to measure changes in surface pressure with the help of a suspended Wilhelmy plate (filter paper of dimensions 10 × 25 mm^2^). The entire system was set inside a transparent glove box. Before each experiment, the trough was cleaned with methanol, ethanol, and ultrapure water to minimize impurities on the surface of the water. PBS was used as the subphase which was maintained at a temperature of 25 °C. Lipid solution of 1 mg/ml concentration was spread gently over the air/water interface until a surface pressure of 2 to 3 mN/m was reached and was left undisturbed for 15 min to relax the monolayer to 0 mN/m. 10 μl of Aβ 40 (23 μM) was injected into the subphase before monolayer compression. The subphase was gently stirred using a magnetic stirrer. Surface pressure(π)-Molecular area(A) Isotherms were recorded by compressing the monolayer at a constant speed of 3 mm/min. Isotherms were recorded until collapse pressure (π_c_) was reached. Isotherm data were used to process the compressibility modulus ($C_{s}^{-1}$) (38, 39, 71, 72).

(7) $$C_{s}^{-1}=-A\frac{d\pi }{dA}$$

and bending force (${\Delta F}_{b}$).

(8) $${\Delta F}_{b}={MM\;\pi }_{c}-{MM\pi }_{c}$$

where MMAβπ_c_ is the collapse pressure of the model membrane with Aβ in subphase and MMπ_c_ being without Aβ. Negative values of (ΔF_b_) suggest bending of monolayer (73).

For constant area measurements (Time v/s Pressure), the lipid monolayer was first compressed to a surface pressure of 30 mN/m and left to relax for 10 min. Aβ 40 peptide was injected from underneath one of the barriers to avoid disturbing the monolayer and subsequently recorded for changes in surface pressure over time.

### Image processing

ImageJ’s oval profile plugin was used to extract intensity data from the circumference of GUVs imaged under a confocal microscope. Tubeness plugin was used to resolve the fibril-like structures present in the fluorescence images of the GUVs. The plugin utilizes the eigenvalues of the Hessian matrix to calculate the measure of “tubeness” in the case of 2D images, if the larger eigenvalue is negative, an absolute value is returned otherwise it is returned as 0.

## Data availability

All the data are available within the main article and the supporting information files.

## Supporting information

This article contains supporting information.

## Conflict of interest

The authors declare that they have no conflicts of interest with the contents of this article.

## References

[1] Kaya I., Jennische E., Lange S., Tarik Baykal A., Malmberg P., Fletcher J.S. (2020). Brain region-specific amyloid plaque-associated myelin lipid loss, APOE deposition and disruption of the myelin sheath in familial Alzheimer’s disease mice. J. Neurochem..

[2] Lehmann S., Dumurgier J., Ayrignac X., Marelli C., Alcolea D., Ormaechea J.F. (2020). Cerebrospinal fluid A beta 1–40 peptides increase in Alzheimer’s disease and are highly correlated with phospho-tau in control individuals. Alzheimers Res. Ther..

[3] Collins-Praino L.E., Francis Y.I., Griffith E.Y., Wiegman A.F., Urbach J., Lawton A. (2014). Soluble amyloid beta levels are elevated in the white matter of Alzheimer’s patients, independent of cortical plaque severity. Acta Neuropathol. Commun..

[4] Tse K.-H., Cheng A., Ma F., Herrup K. (2018). DNA damage-associated oligodendrocyte degeneration precedes amyloid pathology and contributes to Alzheimer's disease and dementia. Alzheimers Dement..

[5] Ferreira S., Pitman K.A., Wang S., Summers B.S., Bye N., Young K.M. (2020). Amyloidosis is associated with thicker myelin and increased oligodendrogenesis in the adult mouse brain. J. Neurosci. Res..

[6] Dean D.C., Hurley S.A., Kecskemeti S.R., O'Grady J.P., Canda C., Davenport-Sis N.J. (2017). Association of amyloid pathology with myelin alteration in preclinical Alzheimer disease. JAMA Neurol..

[7] Sanderson J.M. (2022). The association of lipids with amyloid fibrils. J. Biol. Chem..

[8] Yip C.M., McLaurin J. (2001). Amyloid-β peptide assembly: a critical step in fibrillogenesis and membrane disruption. Biophys. J..

[9] Sciacca M.F., Lolicato F., Tempra C., Scollo F., Sahoo B.R., Watson M.D. (2020). Lipid-chaperone hypothesis: a common molecular mechanism of membrane disruption by intrinsically disordered proteins. ACS Chem. Neurosci..

[10] Korshavn K.J., Satriano C., Lin Y., Zhang R., Dulchavsky M., Bhunia A. (2017). Reduced lipid bilayer thickness regulates the aggregation and cytotoxicity of amyloid-β∗. J. Biol. Chem..

[11] Nguyen P.H., Ramamoorthy A., Sahoo B.R., Zheng J., Faller P., Straub J.E. (2021). Amyloid oligomers: a joint experimental/computational perspective on Alzheimer’s disease, Parkinson’s disease, type II diabetes, and amyotrophic lateral sclerosis. Chem. Rev..

[12] Eckert A., Hauptmann S., Scherping I., Meinhardt J., Rhein V., Dröse S. (2008). Oligomeric and fibrillar species of β-amyloid (Aβ42) both impair mitochondrial function in P301L tau transgenic mice. J. Mol. Med..

[13] Tian Y., Liang R., Kumar A., Szwedziak P., Viles J.H. (2021). 3D-visualization of amyloid-β oligomer interactions with lipid membranes by cryo-electron tomography. Chem. Sci..

[14] Sciacca M.F., Tempra C., Scollo F., Milardi D., La Rosa C. (2018). Amyloid growth and membrane damage: current themes and emerging perspectives from theory and experiments on Aβ and hIAPP. Biochim. Biophys. Acta Biomembr..

[15] Lauwers E., Goodchild R., Verstreken P. (2016). Membrane lipids in presynaptic function and disease. Neuron.

[16] Shrivastava A.N., Aperia A., Melki R., Triller A. (2017). Physico-pathologic mechanisms involved in neurodegeneration: misfolded protein-plasma membrane interactions. Neuron.

[17] Cohen S.I., Linse S., Luheshi L.M., Hellstrand E., White D.A., Rajah L. (2013). Proliferation of amyloid-β42 aggregates occurs through a secondary nucleation mechanism. Proc. Natl. Acad. Sci. U. S. A..

[18] Calderón R.O., DeVries G.H. (1997). Lipid composition and phospholipid asymmetry of membranes from a Schwann cell line. J. Neurosci. Res..

[19] Fitzner D., Bader J.M., Penkert H., Bergner C.G., Su M., Weil M.-T. (2020). Cell-type- and brain-region-resolved mouse brain lipidome. Cell Rep..

[20] Bode D.C., Freeley M., Nield J., Palma M., Viles J.H. (2019). Amyloid-β oligomers have a profound detergent-like effect on lipid membrane bilayers, imaged by atomic force and electron microscopy. J. Biol. Chem..

[21] Rodi P.M., Maggio B., Bagatolli L.A. (2018). Direct visualization of the lateral structure of giant vesicles composed of pseudo-binary mixtures of sulfatide, asialo-GM1 and GM1 with POPC. Biochim. Biophys. Acta Biomembr..

[22] Sowade R.F., Jahn T.R. (2017). Seed-induced acceleration of amyloid-β mediated neurotoxicity *in vivo*. Nat. Commun..

[23] Kinoshita M., Kakimoto E., Terakawa M.S., Lin Y., Ikenoue T., So M. (2017). Model membrane size-dependent amyloidogenesis of Alzheimer's amyloid-β peptides. Phys. Chem. Chem. Phys..

[24] McMahon H.T., Boucrot E. (2015). Membrane curvature at a glance. J. Cell. Sci..

[25] Strandberg E., Tiltak D., Ehni S., Wadhwani P., Ulrich A.S. (2012). Lipid shape is a key factor for membrane interactions of amphipathic helical peptides. Biochim. Biophys. Acta.

[26] Larsen J.B., Kennard C., Pedersen S.L., Jensen K.J., Uline M.J., Hatzakis N.S. (2017). Membrane curvature and lipid composition synergize to regulate N-ras anchor recruitment. Biophys. J..

[27] Bera S., Gayen N., Mohid S.A., Bhattacharyya D., Krishnamoorthy J., Sarkar D. (2020). Comparison of synthetic neuronal model membrane mimics in amyloid aggregation at atomic resolution. ACS Chem. Neurosci..

[28] Gautier R., Bacle A., Tiberti M.L., Fuchs P.F., Vanni S., Antonny B. (2018). PackMem: a versatile tool to compute and visualize interfacial packing defects in lipid bilayers. Biophys. J..

[29] Chakraborty S., Doktorova M., Molugu T.R., Heberle F.A., Scott H.L., Dzikovski B. (2020). How cholesterol stiffens unsaturated lipid membranes. Proc. Natl. Acad. Sci. U. S. A..

[30] Greiner A.J., Pillman H.A., Worden R., Blanchard G., Ofoli R. (2009). Effect of hydrogen bonding on the rotational and translational dynamics of a headgroup-bound chromophore in bilayer lipid membranes. J. Phys. Chem. B.

[31] Milowska K., Rodacka A., Melikishvili S., Buczkowski A., Pałecz B., Waczulikova I. (2021). Dendrimeric HIV-peptide delivery nanosystem affects lipid membranes structure. Sci. Rep..

[32] Choucair A., Chakrapani M., Chakravarthy B., Katsaras J., Johnston L. (2007). Preferential accumulation of Aβ (1− 42) on gel phase domains of lipid bilayers: an AFM and fluorescence study. Biochim. Biophys. Acta.

[33] Krausser J., Knowles T.P., Šarić A. (2020). Physical mechanisms of amyloid nucleation on fluid membranes. Proc. Natl. Acad. Sci. U. S. A..

[34] Yang S.-T., Kiessling V., Tamm L.K. (2016). Line tension at lipid phase boundaries as driving force for HIV fusion peptide-mediated fusion. Nat. Commun..

[35] Chi E.Y., Ege C., Winans A., Majewski J., Wu G., Kjaer K. (2008). Lipid membrane templates the ordering and induces the fibrillogenesis of Alzheimer's disease amyloid-β peptide. Proteins.

[36] Stefaniu C., Brezesinski G., Möhwald H. (2014). Langmuir monolayers as models to study processes at membrane surfaces. Adv. Colloid Interface Sci..

[37] Broniatowski M., Flasiński M., Dynarowicz-tka P., Majewski J. (2010). Grazing incidence diffraction and X-ray reflectivity studies of the interactions of inorganic mercury salts with membrane lipids in Langmuir monolayers at the air/water interface. J. Phys. Chem. B.

[38] Brockman H. (1999). Lipid monolayers: why use half a membrane to characterize protein-membrane interactions?. Curr. Opin. Struct. Biol..

[39] Brown R.E., Brockman H.L., McIntosh T.J. (2007). Lipid Rafts.

[40] De S., Wirthensohn D.C., Flagmeier P., Hughes C., Aprile F.A., Ruggeri F.S. (2019). Different soluble aggregates of Aβ42 can give rise to cellular toxicity through different mechanisms. Nat. Commun..

[41] Saleem M., Morlot S., Hohendahl A., Manzi J., Lenz M., Roux A. (2015). A balance between membrane elasticity and polymerization energy sets the shape of spherical clathrin coats. Nat. Commun..

[42] Morlot S., Galli V., Klein M., Chiaruttini N., Manzi J., Humbert F. (2012). Membrane shape at the edge of the dynamin helix sets location and duration of the fission reaction. Cell.

[43] Van Hilten N., Stroh K.S., Risselada H.J. (2020). Membrane thinning induces sorting of lipids and the amphipathic lipid packing sensor (ALPS) protein motif. Front. Physiol..

[44] Lindberg D.J., Wesén E., Björkeroth J., Rocha S., Esbjörner E.K. (2017). Lipid membranes catalyse the fibril formation of the amyloid-β (1–42) peptide through lipid-fibril interactions that reinforce secondary pathways. Biochim. Biophys. Acta Biomembr..

[45] Yi X., Zhang Y., Gong M., Yu X., Darabedian N., Zheng J. (2015). Ca2+ interacts with Glu-22 of Aβ (1–42) and phospholipid bilayers to accelerate the Aβ (1–42) aggregation below the critical micelle concentration. Biochemistry.

[46] Sciacca M.F., Brender J.R., Lee D.-K., Ramamoorthy A. (2012). Phosphatidylethanolamine enhances amyloid fiber-dependent membrane fragmentation. Biochemistry.

[47] McLaurin J., Franklin T., Chakrabartty A., Fraser P. (1998). Phosphatidylinositol and inositol involvement in Alzheimer amyloid-β fibril growth and arrest. J. Mol. Biol..

[48] Martins I.C., Kuperstein I., Wilkinson H., Maes E., Vanbrabant M., Jonckheere W. (2008). Lipids revert inert Aβ amyloid fibrils to neurotoxic protofibrils that affect learning in mice. EMBO J..

[49] Engel M.F., Khemtémourian L., Kleijer C.C., Meeldijk H.J., Jacobs J., Verkleij A.J. (2008). Membrane damage by human islet amyloid polypeptide through fibril growth at the membrane. Proc. Natl. Acad. Sci. U. S. A..

[50] Butterfield S.M., Lashuel H.A. (2010). Amyloidogenic protein–membrane interactions: mechanistic insight from model systems. Angew. Chem. Int. Ed. Engl..

[51] Vanni S., Hirose H., Barelli H., Antonny B., Gautier R. (2014). A sub-nanometre view of how membrane curvature and composition modulate lipid packing and protein recruitment. Nat. Commun..

[52] Bigay J., Antonny B. (2012). Curvature, lipid packing, and electrostatics of membrane organelles: defining cellular territories in determining specificity. Dev. Cell.

[53] Zhang X., St. Clair J.R., London E., Raleigh D.P. (2017). Islet amyloid polypeptide membrane interactions: effects of membrane composition. Biochemistry.

[54] Yang Y., Jalali S., Nilsson B.L., Dias C.L. (2021). Binding mechanisms of amyloid-like peptides to lipid bilayers and effects of divalent cations. ACS Chem. Neurosci..

[55] Ji S.-R., Wu Y., Sui S.-f. (2002). Cholesterol is an important factor affecting the membrane insertion of β-amyloid peptide (Aβ1–40), which may potentially inhibit the fibril formation. J. Biol. Chem..

[56] Yu X., Zheng J. (2012). Cholesterol promotes the interaction of Alzheimer β-amyloid monomer with lipid bilayer. J. Mol. Biol..

[57] Iyer A., Schilderink N., Claessens M.M.A.E., Subramaniam V. (2016). Membrane-bound alpha synuclein clusters induce impaired lipid diffusion and increased lipid packing. Biophys. J..

[58] Steinkühler J., Jacobs M.L., Boyd M.A., Villaseñor C.G., Loverde S.M., Kamat N.P. (2022). PEO-b-PBD diblock copolymers induce packing defects in lipid/hybrid membranes and improve insertion rates of natively folded peptides. Biomacromolecules.

[59] Braun A.R., Lacy M.M., Ducas V.C., Rhoades E., Sachs J.N. (2014). α-Synuclein-induced membrane remodeling is driven by binding affinity, partition depth, and interleaflet order asymmetry. J. Am. Chem. Soc..

[60] Xue W.-F., Hellewell A.L., Gosal W.S., Homans S.W., Hewitt E.W., Radford S.E. (2009). Fibril fragmentation enhances amyloid cytotoxicity. J. Biol. Chem..

[61] Sciacca M.F., Kotler S.A., Brender J.R., Chen J., Lee D.-k., Ramamoorthy A. (2012). Two-step mechanism of membrane disruption by Aβ through membrane fragmentation and pore formation. Biophys. J..

[62] Wong P.T., Schauerte J.A., Wisser K.C., Ding H., Lee E.L., Steel D.G. (2009). Amyloid-β membrane binding and permeabilization are distinct processes influenced separately by membrane charge and fluidity. J. Mol. Biol..

[63] Ding H., Schauerte J.A., Steel D.G., Gafni A. (2012). β-Amyloid (1–40) peptide interactions with supported phospholipid membranes: a single-molecule study. Biophys. J..

[64] Baumgart T., Hammond A.T., Sengupta P., Hess S.T., Holowka D.A., Baird B.A. (2007). Large-scale fluid/fluid phase separation of proteins and lipids in giant plasma membrane vesicles. Proc. Natl. Acad. Sci. U. S. A..

[65] Weinberger A., Tsai F.-C., Koenderink G.H., Schmidt T.F., Itri R., Meier W. (2013). Gel-assisted formation of giant unilamellar vesicles. Biophys. J..

[66] Tiwari A., Prince A., Arakha M., Jha S., Saleem M. (2018). Passive membrane penetration by ZnO nanoparticles is driven by the interplay of electrostatic and phase boundary conditions. Nanoscale.

[67] Marrink S.J., Risselada H.J., Yefimov S., Tieleman D.P., De Vries A.H. (2007). The MARTINI force field: coarse grained model for biomolecular simulations. J. Phys. Chem. B.

[68] Abraham M.J., Murtola T., Schulz R., Páll S., Smith J.C., Hess B. (2015). Gromacs: high performance molecular simulations through multi-level parallelism from laptops to supercomputers. SoftwareX.

[69] Williams T., Kelley C., Bröker H.-B., Campbell J., Cunningham R., Denholm D. (1986). An interactive plotting program. Environment.

[70] Chattopadhyay K., Saffarian S., Elson E.L., Frieden C. (2002). Measurement of microsecond dynamic motion in the intestinal fatty acid binding protein by using fluorescence correlation spectroscopy. Proc. Natl. Acad. Sci. U. S. A..

[71] Allende D., Vidal A., McIntosh T.J. (2004). Jumping to rafts: gatekeeper role of bilayer elasticity. Trends Biochem. Sci..

[72] Smaby J.M., Kulkarni V.S., Momsen M., Brown R.E. (1996). The interfacial elastic packing interactions of galactosylceramides, sphingomyelins, and phosphatidylcholines. Biophys. J..

[73] Peetla C., Jin S., Weimer J., Elegbede A., Labhasetwar V. (2014). Biomechanics and thermodynamics of nanoparticle interactions with plasma and endosomal membrane lipids in cellular uptake and endosomal escape. Langmuir.

