# Supplementary material for: Interplay of lipid head group and packing defects in driving amyloid-beta–mediated myelin-like model membrane deformation
Source: J Biol Chem. 2023 Mar 27;299(5):104653. doi: 10.1016/j.jbc.2023.104653 (PMC10148160; doi:10.1016/j.jbc.2023.104653)
Supplement: Supporting information [file mmc7.docx]

Supplementary Information

“Interplay of lipid-head group and packing defects in driving Amyloid-beta mediated myelin-like model membrane deformation”

Anuj Tiwari^1^, Sweta Pradhan^2^, Achinta Sannigrahi^3^, Amaresh Kumar Mahakud^4,6^, Suman Jha^1^, Krishnananda Chattopadhyay^3^, Mithun Biswas^2^, Mohammed Saleem^4,5,6^*

^1^ Department of Life Sciences, National Institute of Technology, Rourkela, India.

^2^ Department of Physics and Astronomy, National Institute of Technology, Rourkela, India.

^3^ CSIR - Indian Institute of Chemical Biology, Kolkata, India

^4^ School of Biological Sciences, National Institute of Science Education and Research, Bhubaneswar, India.

^5^ Centre for Interdisciplinary Sciences, National Institute of Science Education and Research, Bhubaneswar, India.

^6^ Homi Bhabha National Institute, Mumbai, India.

* Corresponding author: [saleem@niser.ac.in](mailto:saleem@niser.ac.in)

**
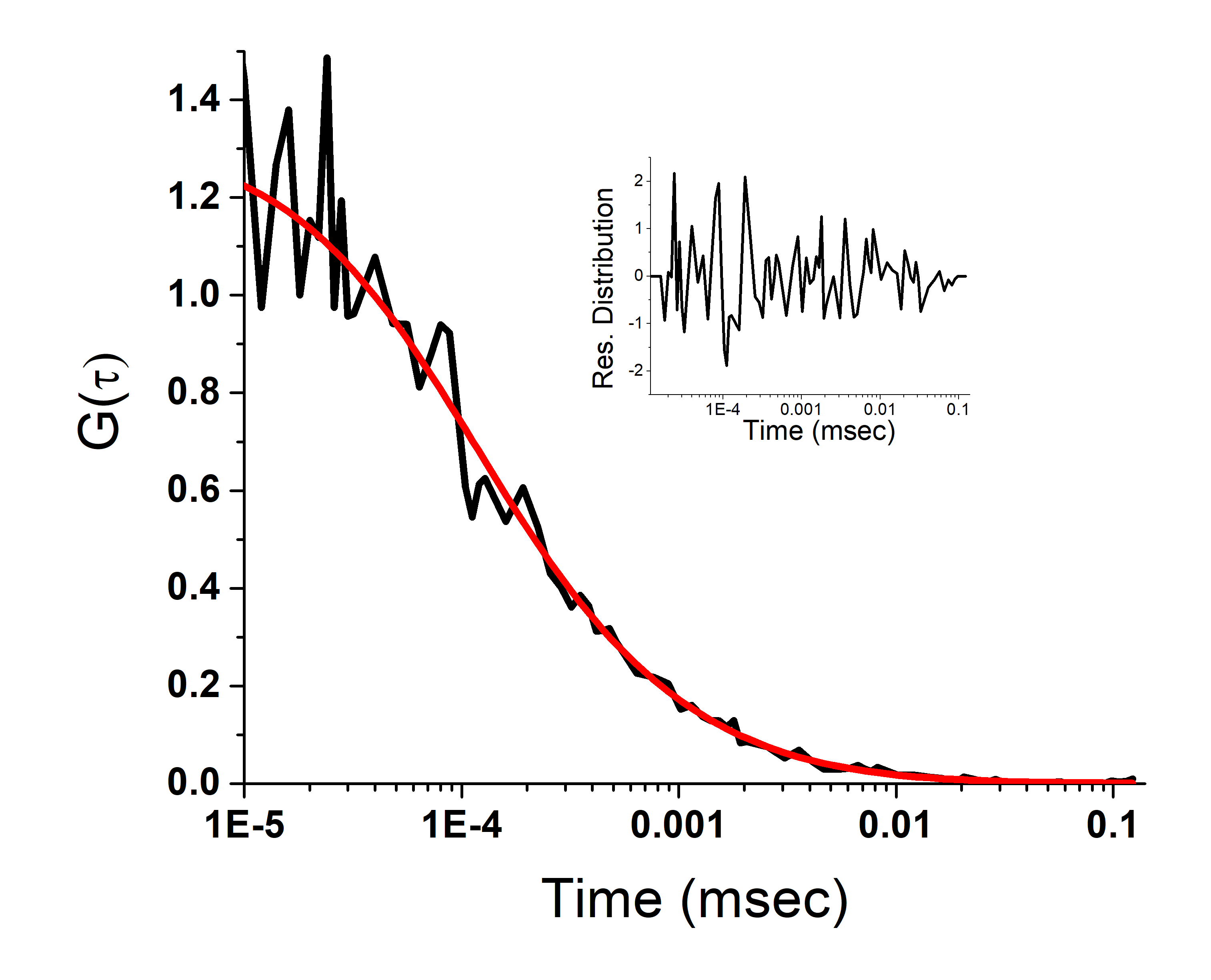
**

| Parameters | |
| --- | --- |
| *D1, mm^2/s* | 268 V |
| *F1* | 0.89 V |
| *D2, mm^2/s* | 29.9 V |
| *C, nM* | 5.52 V |
| *Flow1, mm/s* | 0 F |
| *Flow2, mm/s* | 0 F |
| *w0, mm* | 0.25 F |
| *z0, mm* | 1.25 F |
| *TauD1, ms* | 58.3 |
| *TauD2, ms* | 2.09E+04 |
| *F2* | 0.11 |
| *N, #* | 0.509 |
| *G* | 0.232 |
| *G0* | 0.246 |
| *ExcVol,mm^3* | 0.154 |
| *CPS* | 0 |
| *Chi-Sq* | 0.826 |
| *R_h_ 1 Å* | 9.27 |
| *R_h_ 2 Å* | 83.2 |

**Fig. S1** Fluorescence correlation spectroscopy (FCS) measurements to screen the population of Aβ-40 species present in the sample at a 0-hour time point.


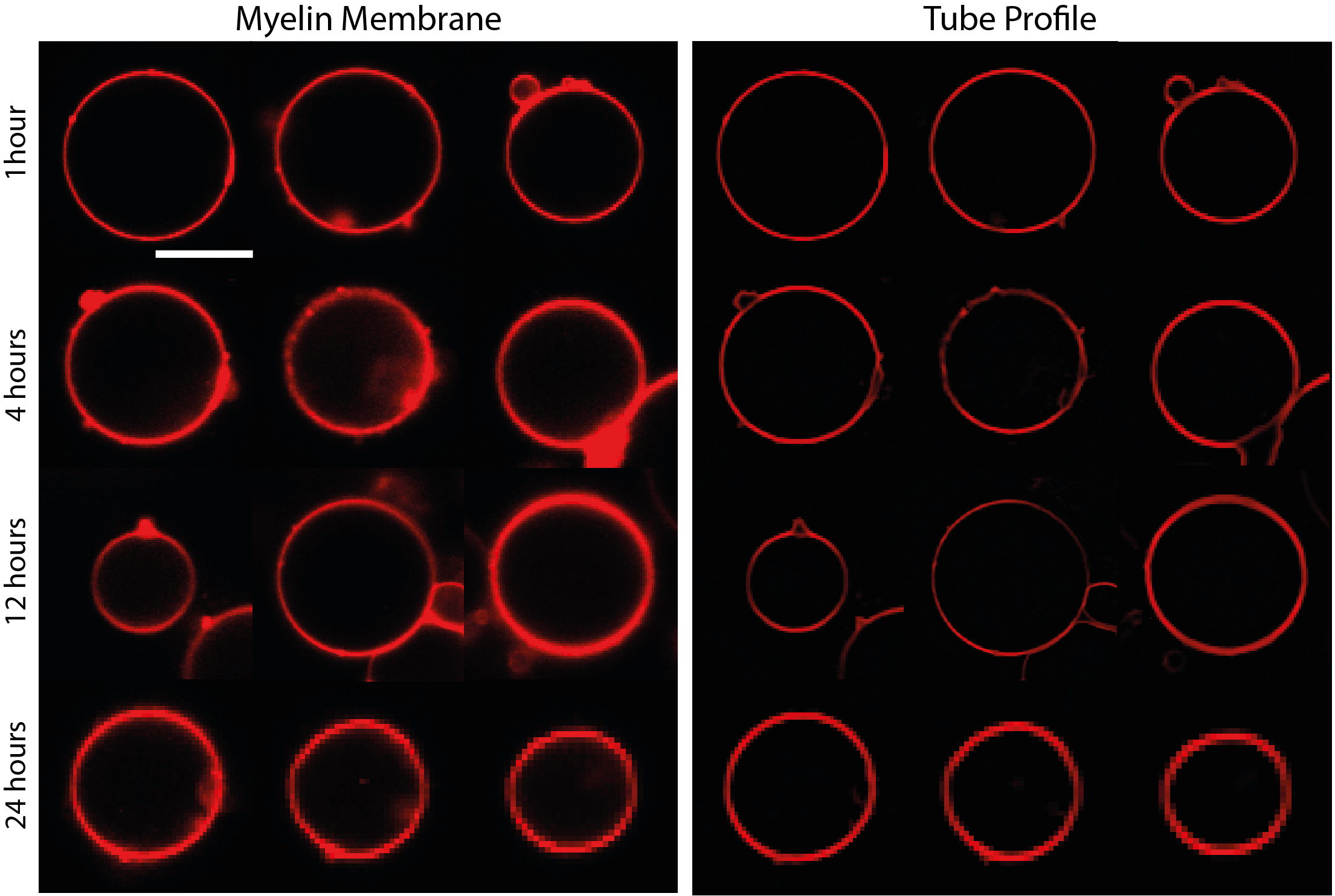

**Fig. S2** **a)** Representative images of GUVs of myelin-like model membrane without Aβ-40 at respective time points. Scale bar is 10 μm. **b)** Supported lipid bilayer of the myelin-like model showing Aβ-40 induced tubular deformations around 8-12 hour onwards. Scale bar is 50 μm.


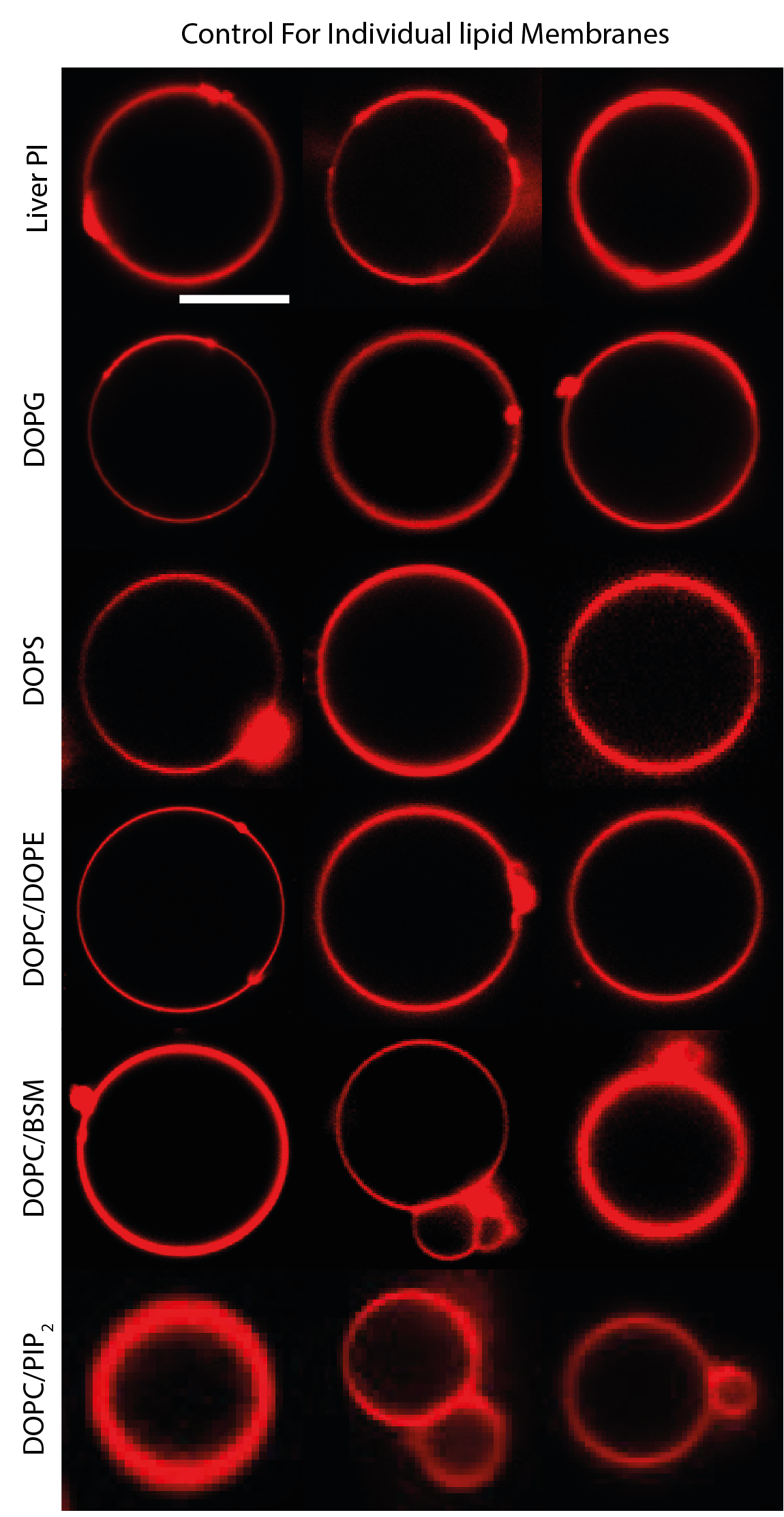


**Fig. S3** Representative images of GUVs of different membrane conditions at a 1-hour time point. Scale bar is 10 μm.


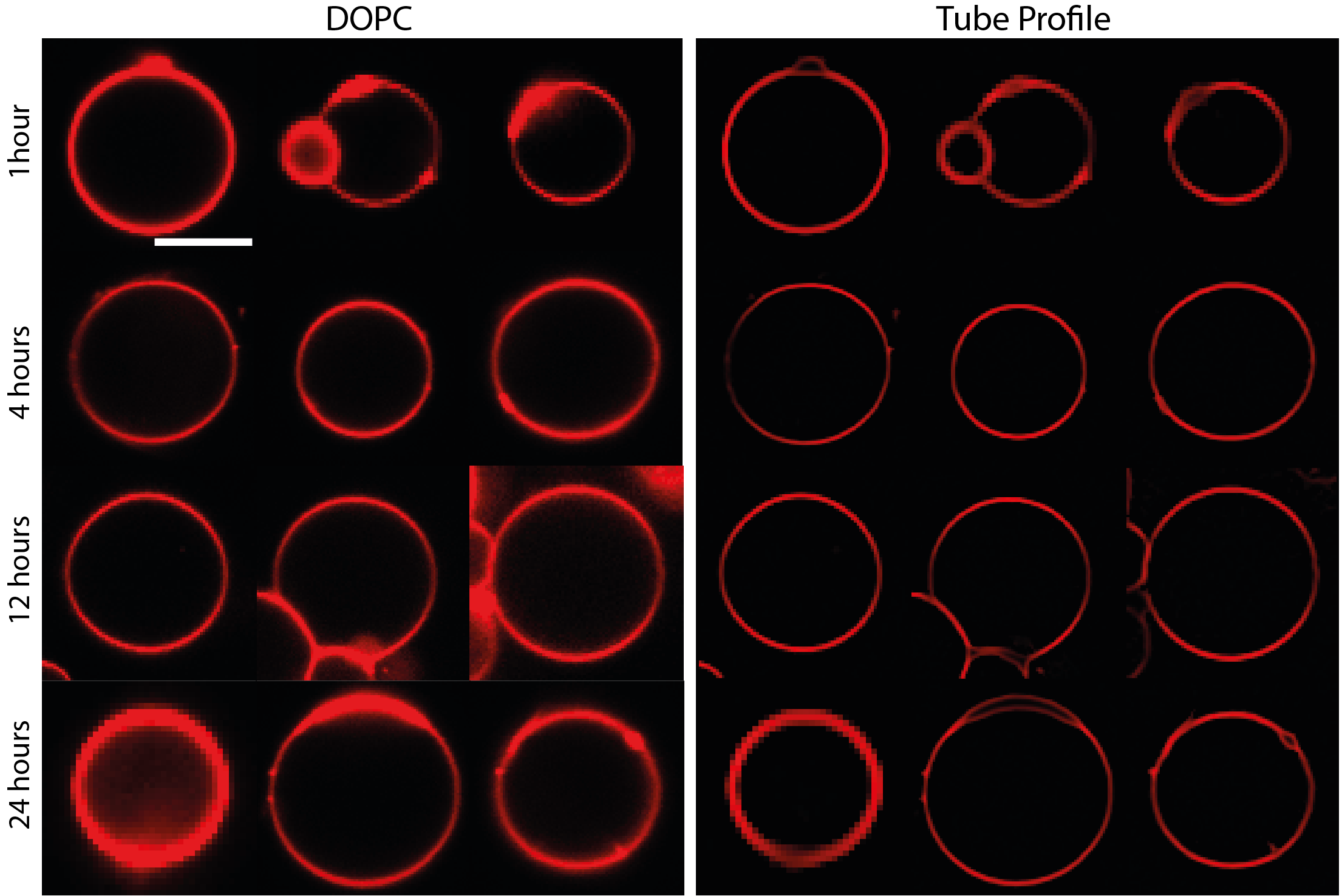


**Fig. S4** Representative images of GUVs of DOPC membrane without Aβ-40 at respective time points. Scale bar is 10 μm.


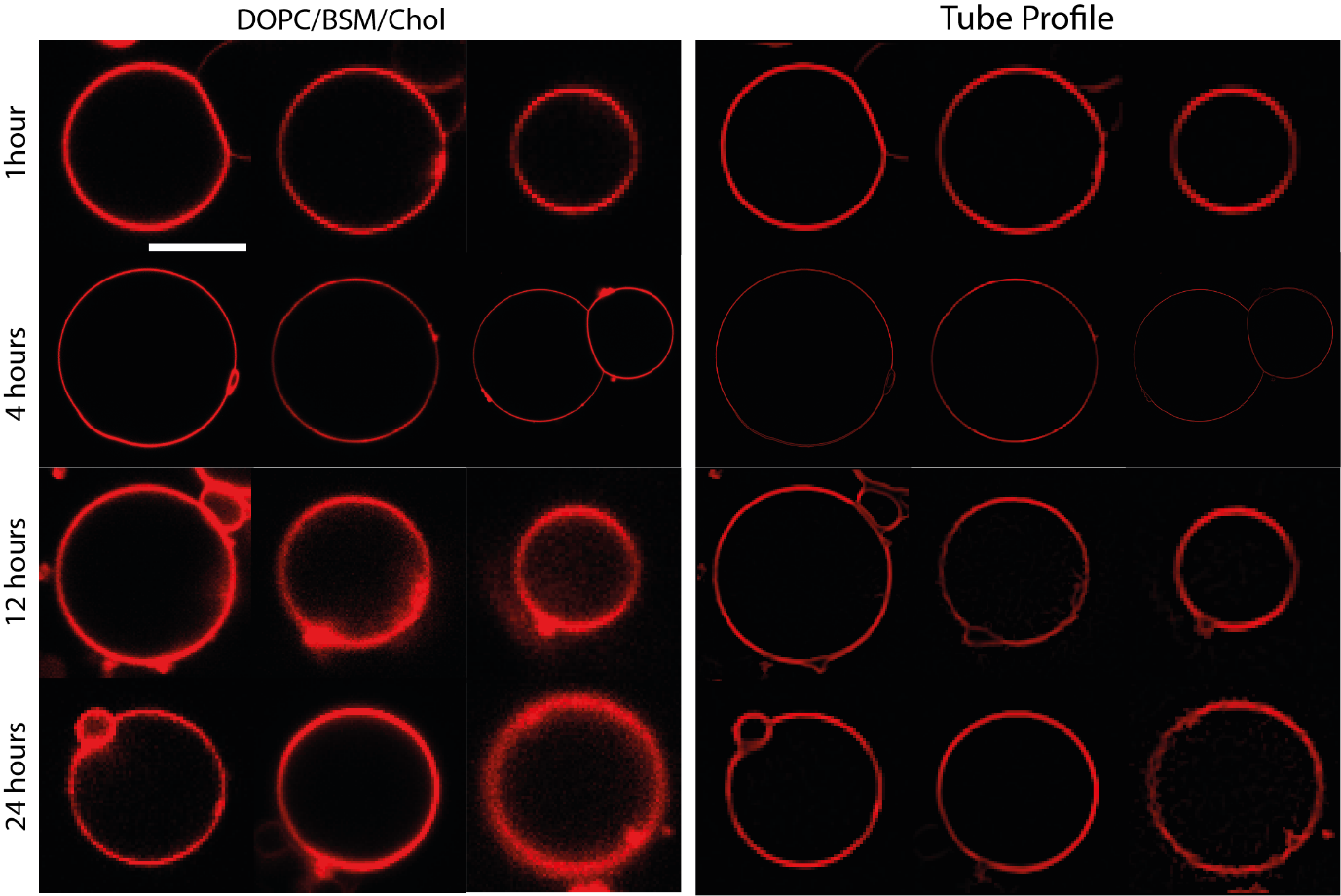


**Fig. S5** Representative images of GUVs of DOPC/BSM/Chol (4:4:2) membrane without Aβ-40 at respective time points. Scale bar is 10 μm.


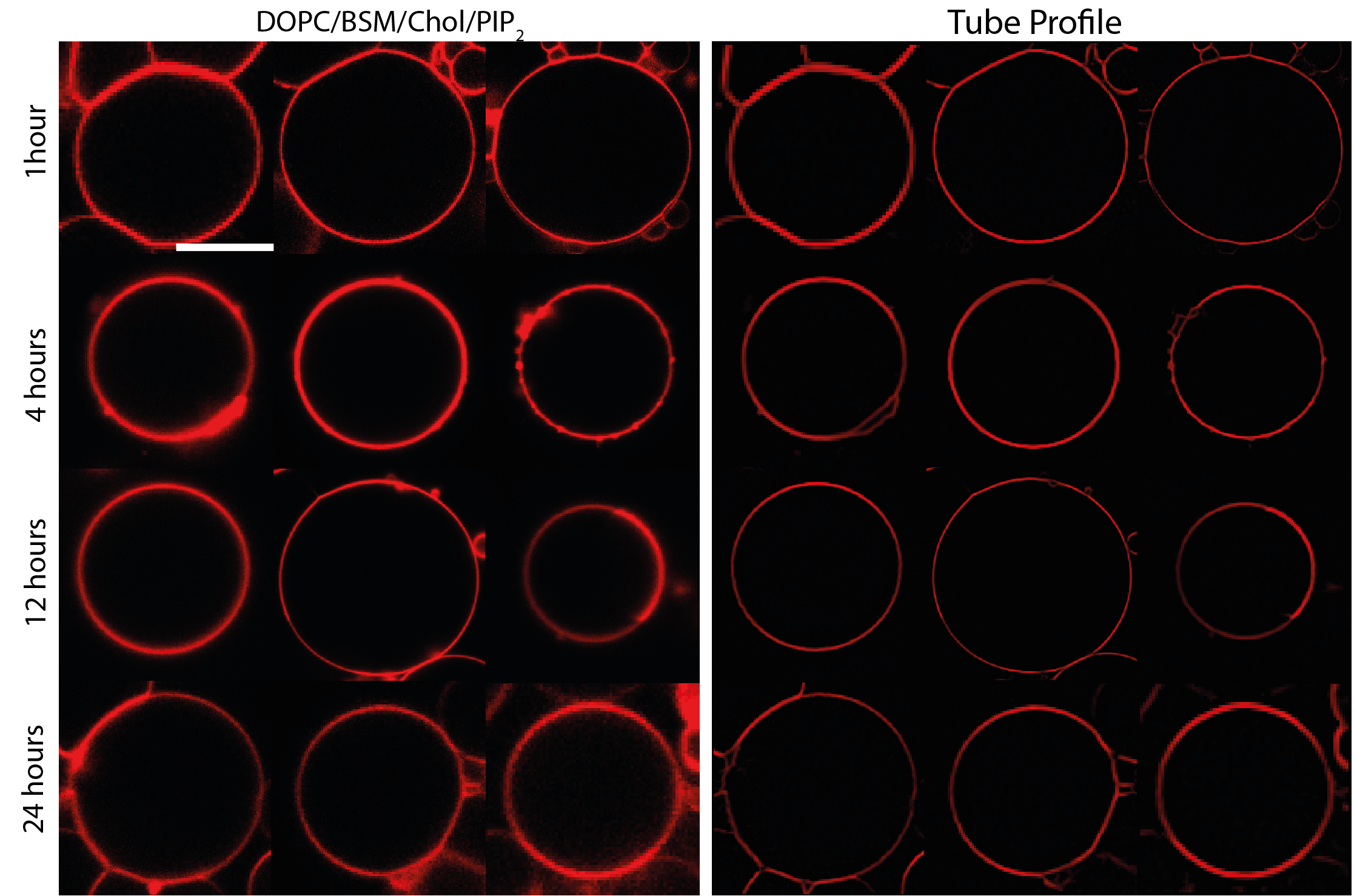


**Fig. S6** Representative images of GUVs of DOPC/BSM/Chol/PIP_2_ (2:4:3:1) membrane without Aβ-40 at respective time points. Scale bar is 10 μm.


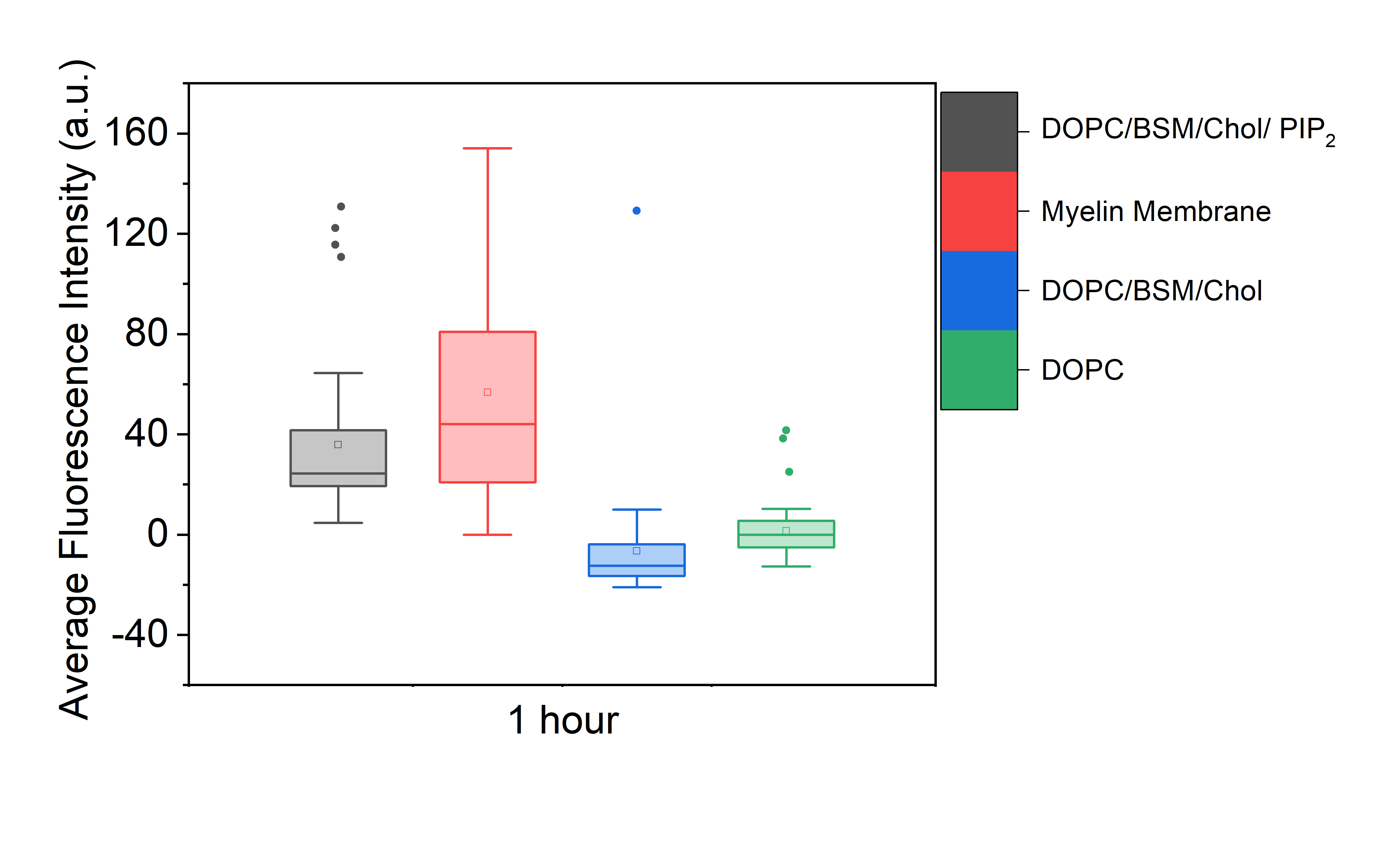


**Fig. S7** Box plot of the average binding intensity of Aβ-40 at the 1-hour time point for different membrane conditions.


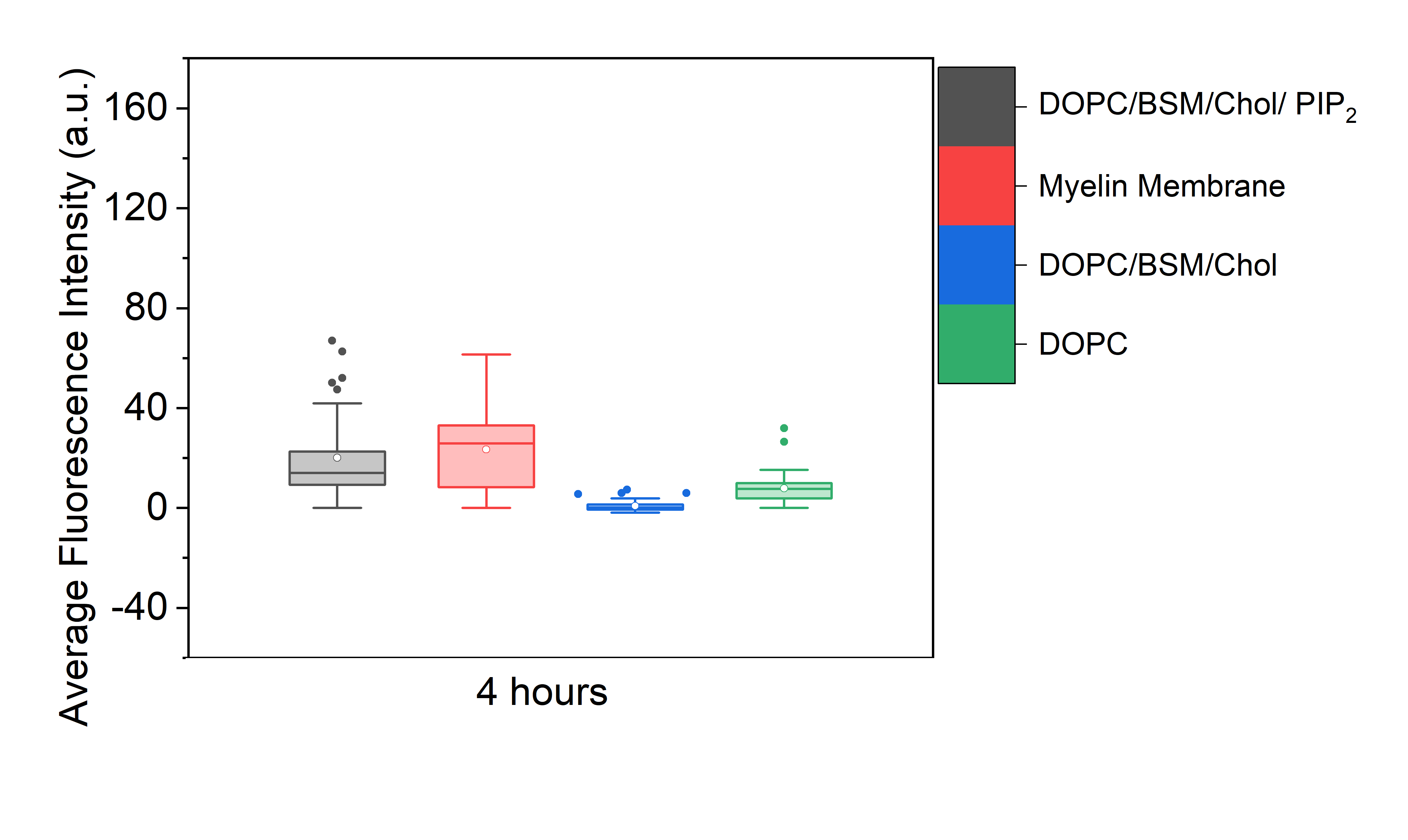


**Fig. S8** Box plot of the average binding intensity of Aβ-40 at the 4-hour time point for different membrane conditions.


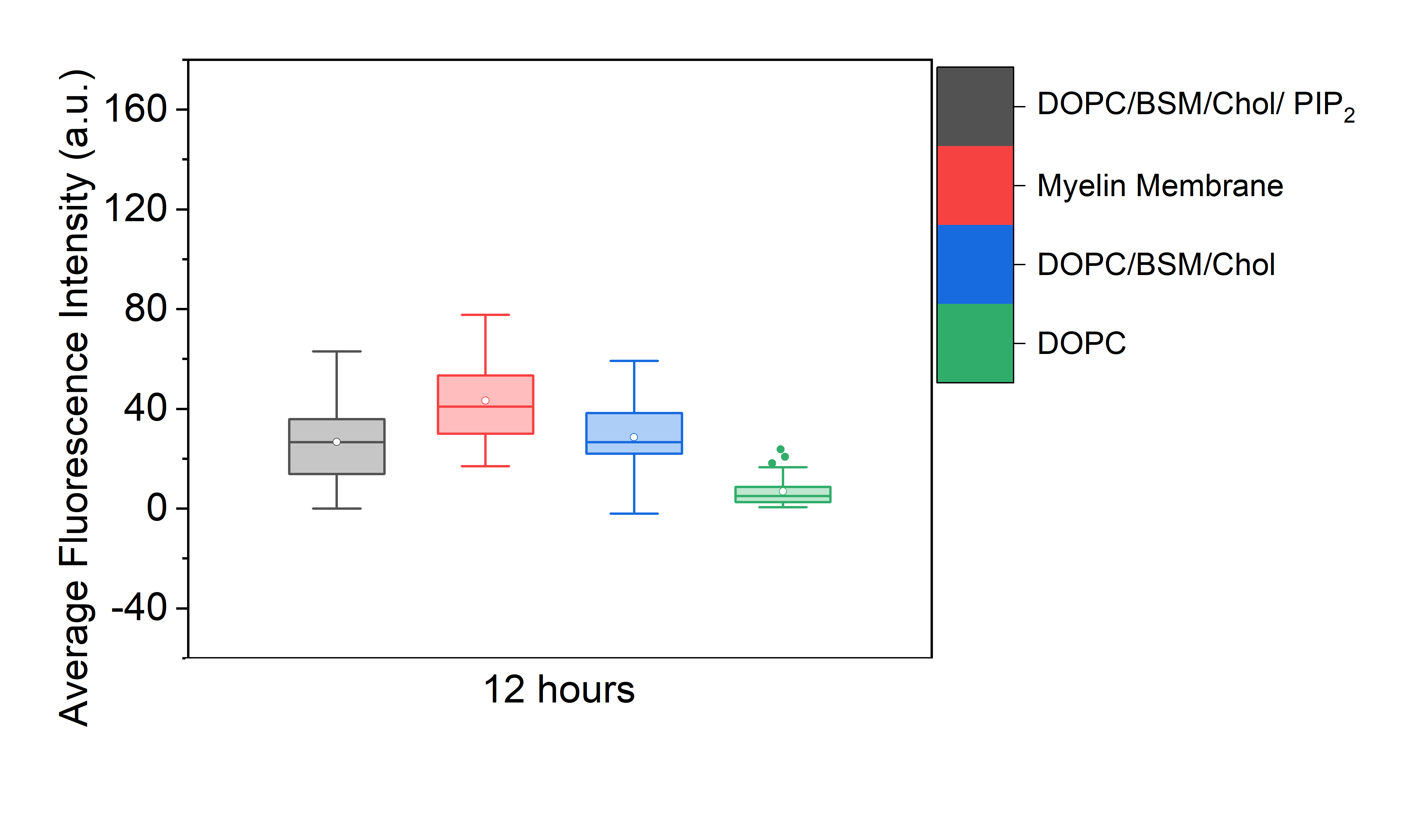


**Fig. S9** Box plot of the average binding intensity of Aβ-40 at the 12-hour time point for different membrane conditions.


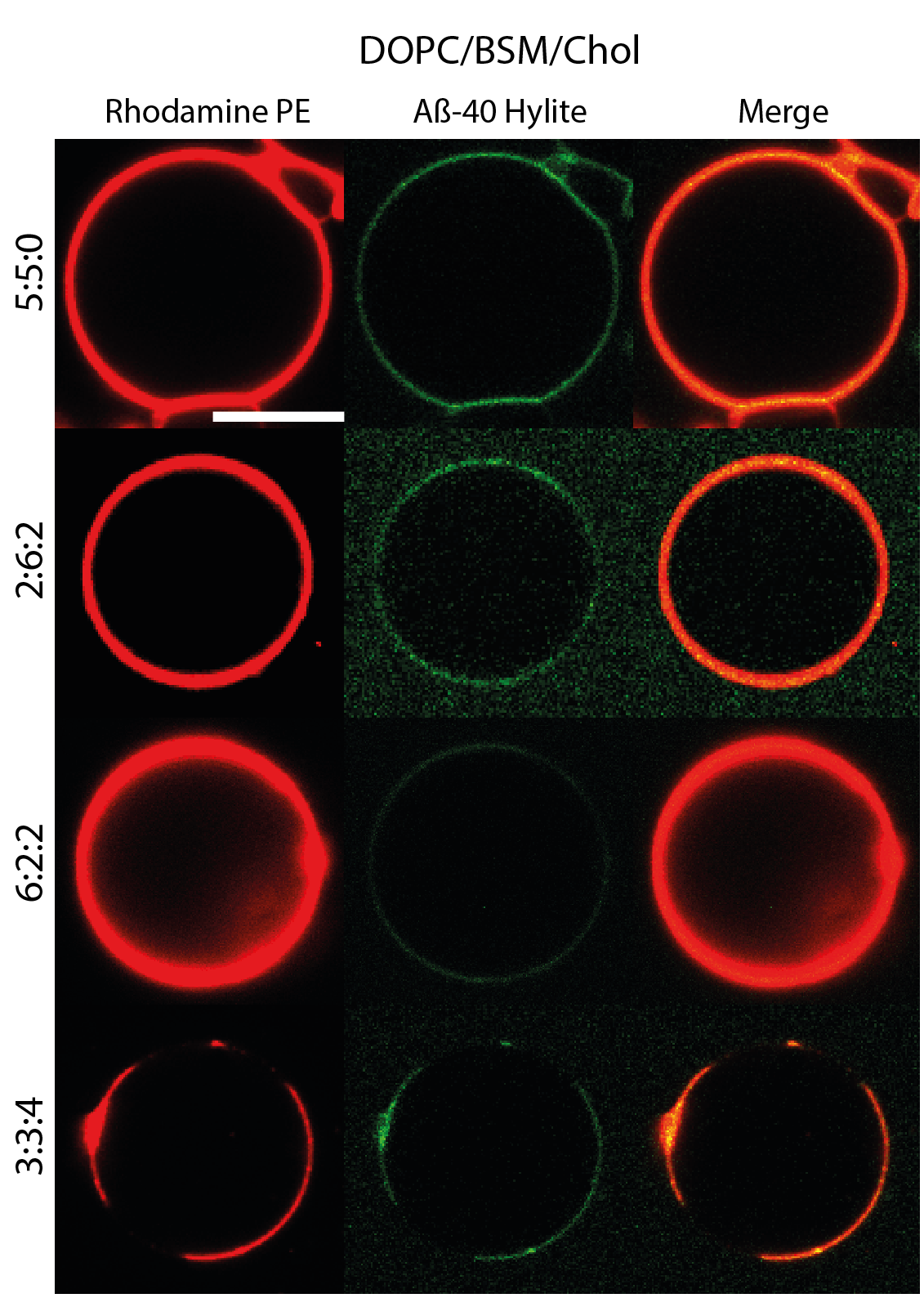


**Fig. S10** Interplay of ratio of lipid shape in a ternary membrane condition containing cholesterol on amyloid binding. Scale bar is 10 μm.


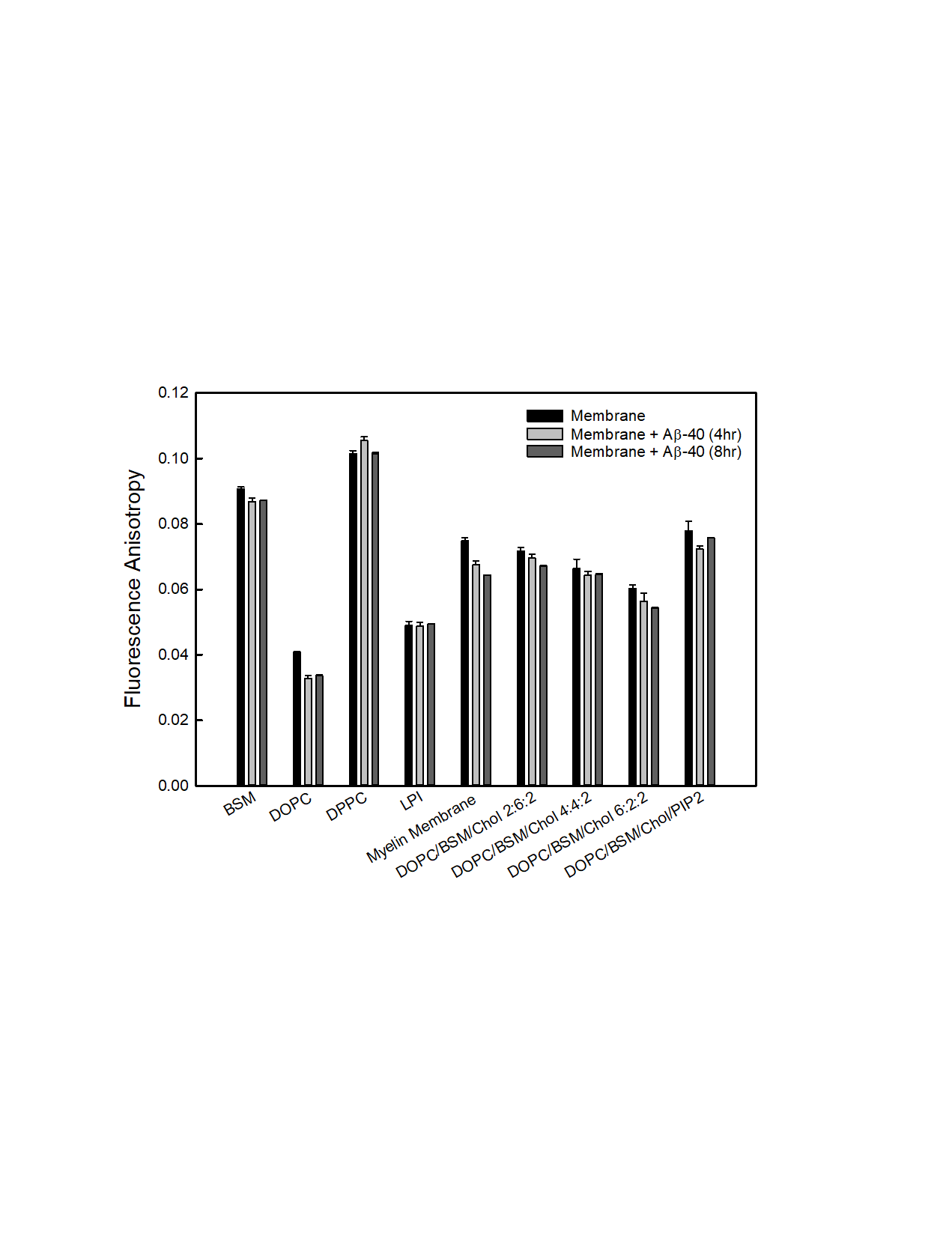


**Fig. S11** Steady-state anisotropy change of TMA-DPH-probed membranes in the absence and presence of amyloid beta over a span of 8 hours. The bar graph represents the mean and standard deviation extracted from three independent experiments.


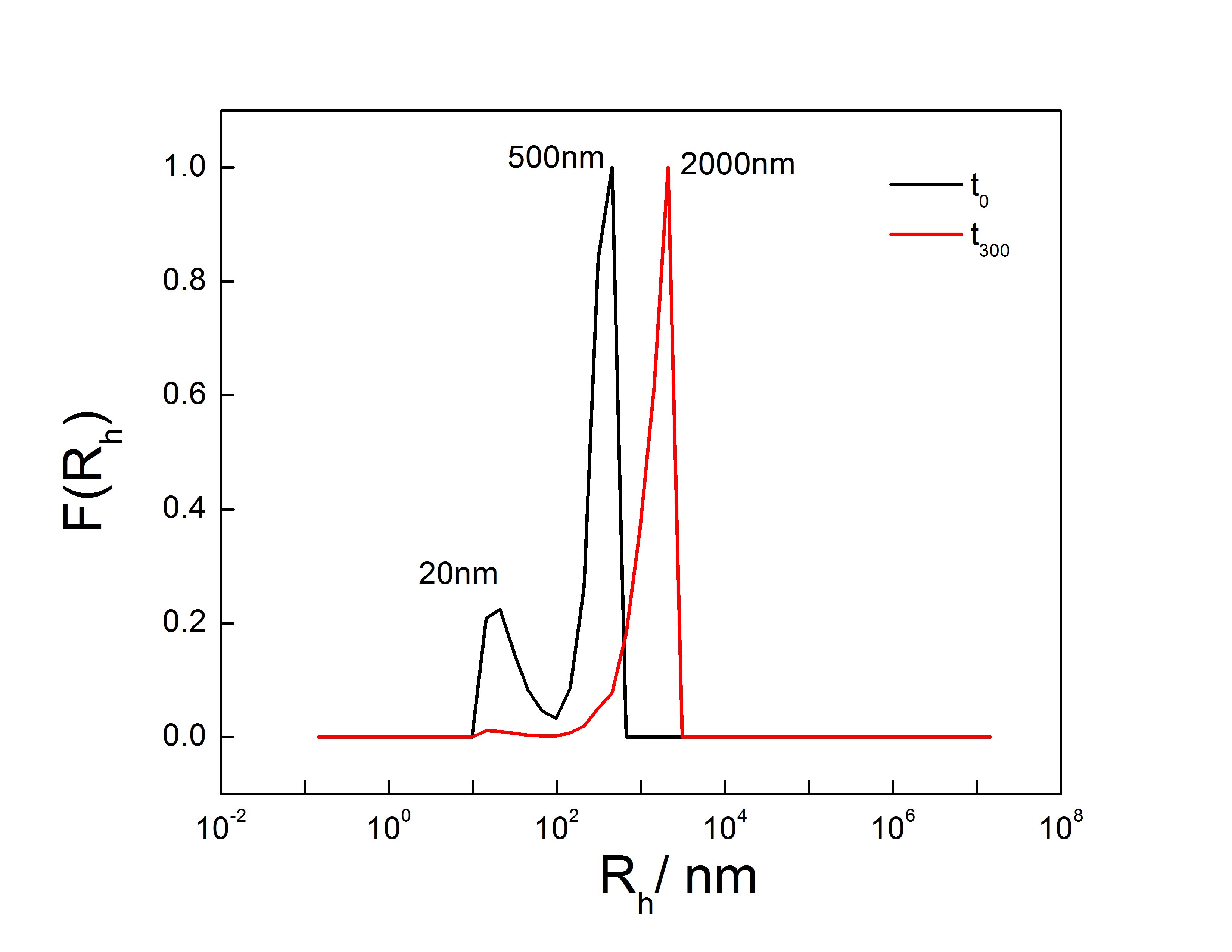


Hydrodynamic Radius Distribution

**Fig. S12** Dynamic light scattering measurement of DOPC LUVs incubated with Aβ-40 showed an increase in size at a 5-hour time point, indicating LUV-LUV fusion.

**Table S1** FCS Table

| Time (*hours*) | D1 *(mm^2^/s)* | SD | D2 *(mm^2^/s)* | SD | TD1 (*ms)* | SD | TD2 (*ms)* | SD | F1 | SD | F2 | SD |
| --- | --- | --- | --- | --- | --- | --- | --- | --- | --- | --- | --- | --- |
| 0 | 225.305 | 61.44050822 | 26.185 | 5.2538034 | 71.87 | 19.1909 | 19839.5 | 1216.93 | 0.85 | 0.0495 | 0.14 | 0.04243 |
| 4 | 375.58 | 10.77630735 | 54.06 | 0.5656854 | 41.61 | 1.19501 | 23142 | 1.41421 | 0.67 | 0.00707 | 0.32 | 0.00707 |
| 8 | 334.575 | 6.201326471 | 56.56 | 2.0081833 | 46.705 | 0.86974 | 24636 | 469.519 | 0.62 | 0 | 0.37 | 0.00707 |

**Table S2** FRAP Diffusion Table

| **Diffusion in µm^2^/s** | | **12 hours** | **24 Hours** |
| --- | --- | --- | --- |
| **Green channel** | DOPC/BSM/Chol | 0.14 | 0.11 |
|  | DOPC/BSM/Chol/PIP2 | 0.29 | 0.17 |
|  | Myelin-like model membrane | 0.23 | 0.23 |
| **Red Channel** | DOPC/BSM/Chol | 0.23 | 0.19 |
|  | DOPC/BSM/Chol/PIP2 | 0.28 | 0.18 |
|  | Myelin-like model membrane | 0.18 | 0.24 |

**Movie S1** FRAP video of DOPC/BSM/Chol at 12 hours

**Movie S2** FRAP video of DOPC/BSM/Chol/PIP2 at 12 hours

**Movie S3** FRAP video of myelin-like model membrane at 12 hours

**Movie S4** FRAP video of DOPC/BSM/Chol at 24 hours

**Movie S5** FRAP video of DOPC/BSM/Chol/PIP2 at 24hours

**Movie S6** FRAP video of myelin-like model membrane at 24 hours
